# Role of Multiple Vanadium Centers on Redox Buffering and Rates of Polyvanadomolybdate-Cu(II)-Catalyzed Aerobic Oxidations

**DOI:** 10.1021/acs.inorgchem.3c00469

**Published:** 2023-03-28

**Authors:** Xinlin Lu, Yurii V. Geletii, Ting Cheng, Craig L. Hill

**Affiliations:** Department of Chemistry, Emory University, Atlanta, Georgia 30322, United States

## Abstract

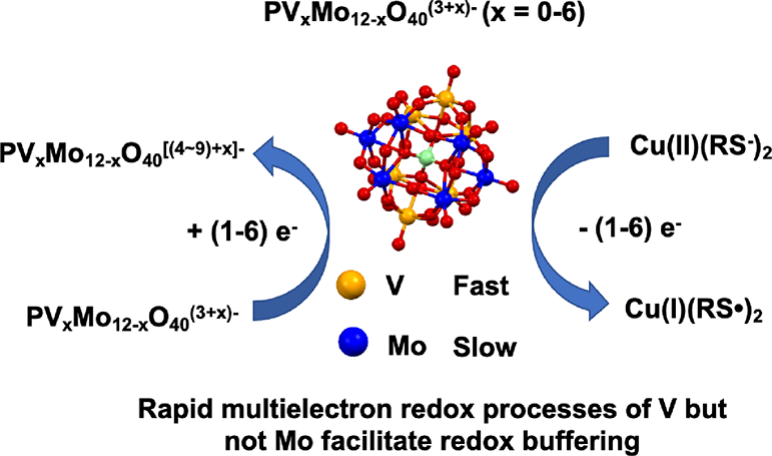

A recent report established that the tetrabutylammonium (TBA) salt of hexavanadopolymolybdate TBA_4_H_5_[PMo_6_V_6_O_40_] (**PV_6_Mo_6_**) serves as the redox buffer with Cu(II) as a co-catalyst for aerobic deodorization of thiols in acetonitrile. Here, we document the profound impact of vanadium atom number (*x* = 0–4 and 6) in TBA salts of PV_*x*_Mo_12–*x*_O_40_^(3+*x*)–^ (**PVMo**) on this multicomponent catalytic system. The **PVMo** cyclic voltammetric peaks from 0 to −2000 mV vs Fc/Fc^+^ under catalytic conditions (acetonitrile, ambient T) are assigned and clarify that the redox buffering capability of the **PVMo**/Cu catalytic system derives from the number of steps, the number of electrons transferred each step, and the potential ranges of each step. All **PVMo** are reduced by varying numbers of electrons, from 1 to 6, in different reaction conditions. Significantly, **PVMo** with *x* ≤ 3 not only has much lower activity than when *x* > 3 (for example, the turnover frequencies (TOF) of **PV_3_Mo_9_** and **PV_4_Mo_8_** are 8.9 and 48 s^–1^, respectively) but also, unlike the latter, cannot maintain steady reduction states when the Mo atoms in these polyoxometalate (POMs) are also reduced. Stopped-flow kinetics measurements reveal that Mo atoms in Keggin **PVMo** exhibit much slower electron transfer rates than V atoms. There are two kinetic arguments: (a) In acetonitrile, the first formal potential of **PMo_12_** is more positive than that of **PVMo_11_** (−236 and −405 mV vs Fc/Fc^+^); however, the initial reduction rates are 1.06 × 10^−4^ s^−1^ and 0.036 s^–1^ for **PMo_12_** and **PVMo_11_**, respectively. (b) In aqueous sulfate buffer (pH = 2), a two-step kinetics is observed for **PVMo_11_** and **PV_2_Mo_10_**, where the first and second steps are assigned to reduction of the V and Mo centers, respectively. Since fast and reversible electron transfers are key for the redox buffering behavior, the slower electron transfer kinetics of Mo preclude these centers functioning in redox buffering that maintains the solution potential. We conclude that **PVMo** with more vanadium atoms allows the POM to undergo more and faster redox changes, which enables the POM to function as a redox buffer dictating far higher catalytic activity.

## Abstract 2

TBA salts of PV_*x*_Mo_12−*x*_O_40_^(3+*x*)−^ (**PVMo**, *x* = 0−6) are multielectron redox buffering complexes that act synergistically with Cu(II) ion to catalyze aerobic thiol oxidation in acetonitrile. The Mo atoms have a much slower electron transfer rate than the V atoms in **PVMo**. As a consequence, the solution electrochemical potential cannot be maintained when the Mo(VI)/M(V) redox couple is involved.

## Introduction

Polyoxometalate (POM) research continues with applications of this large and growing class of complexes in catalysis,^1−5^ biology,^6−10^ environment,^11−14^ and energy.^15−18^ Of particular importance are catalytic O_2_-based oxidations that have been studied over decades with some processes commercialized.^2−5,19,20^ The phosphovanadomolybdates H_3+*x*_PV_*x*_Mo_12–*x*_O_40_^(3+*x*)–^ (*x* = 1–6) have been particularly well studied in context with oxidation of organic substrates.^3,19−21^ This class of POMs can undergo multi-electron transfer^22,23^ and reversible oxygen atom transfer processes^24−30^ and function as radical scavengers.^25,31^ Their oxidations can proceed by different mechanisms including electron transfer (ET),^32,33^ proton-coupled electron transfer (PCET), and electron transfer-oxygen transfer (ET-OT).^24−27^ They function well in Pd(II)-based two-component Wacker-type oxidation reactions.^3,20,34−37^ Noble metal-POM two-component catalytic systems other than those containing Pd(II)^3^ have been studied, and those containing Pt(II) have been reported to oxidize methane.^38,39^ Complementing these studies are reports of POM-stabilized noble-metal(0) nanoparticles catalyzing other oxidation reactions including epoxidation, dehydrogenation, and the conversion of alkanes to alkenes.^40−45^ However, these two-component systems are all based on noble metals. Catalytic systems based on first-row transition metals are desired due to their abundance and low cost. Recently, our group found that Cu(II) catalyzes the reoxidation of reduced Keggin POMs.^46−48^ In addition, a few groups synthesized heteropolyacids (HPAs) with Cu^2+^ counterions and studied their catalytic behaviors.^49−51^ Some studies focused on POMs with different transition metal ions on certain supports as heterogeneous catalysts.^49,51^ Others focused on the effect of metal ion substitution on solution acidity and potential.^50^ In our previous recent work, we found that Cu(II) and the TBA salts of **PV_6_Mo_6_** exhibit high synergy in the air-based oxidation of thiols in acetonitrile.^52^ Unique to this latter study, **PV_6_Mo_6_** behaves as a catalyst for several steps involving multielectron redox buffering,^53,54^ which controls the ratio and speciation of Cu(II)/Cu(I) complexes and maintains the solution potential in narrow ranges based on different POM redox couples.

In this article, we study the effect of number of vanadium atoms, *x*, in TBA salts of phosphovanadomolybdates, PV_*x*_Mo_12–*x*_O_40_^(3+*x*)–^ (**PVMo**, *x* = 0–4 and 6), on the redox buffering properties of the **PVMo**/Cu homogeneous catalytic system for aerobic thiol (RSH) oxidation (eq 1). To do this, we measure the potentials and the number of electrons in the reduced **PVMo** intermediates as well as the greatly different ET rates for V versus Mo in the POMs and show how these properties correlate with catalytic activity and redox self-buffering.
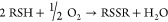
1

## Results and Discussion

**Figure 1:**
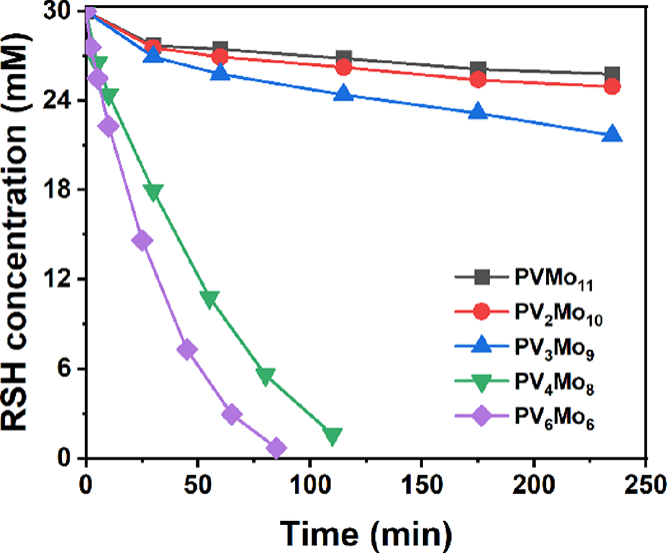
RSH consumption catalyzed by **PVMo**/Cu under air at room temperature. Conditions: POM: 0.1 mM, Cu(II): 0.8 mM, RSH: 30 mM in 5 mL acetonitrile.

The kinetics of RSH consumption catalyzed by the **PVMo**/Cu system are shown in Figure 1. The activity of **PVMo** decreases as the number of vanadium centers decreases from *x* = 6 to *x* = 1. We note that the **PVMo** where *x* = 4 and 6 are dramatically more active than for **PVMo**, where *x* ≤ 3 (data summarized in Table S6). The effect of the substituted vanadium addenda atoms on catalytic activity has been documented in several studies.^32,50,51,55−58^ Two mechanisms have been advanced to explain the trend in activity. The first involves the intact POM polyanion as the catalytically active unit, and the second involves vanadate, VO_2_^+^, that has dissociated from the polyanion, as the active unit.^32,50,59,60^ In the former case, the activity is controlled by the V(V/VI) redox potential and POM structure.^55,57^ We note that in some H_2_O_2_-based oxidations catalyzed by divanadium-substituted polyoxotungstates, peroxo-vanadium intermediates have been proposed.^61,62^ In our previous work,^52^ we conducted a series of experiments to show that free vanadium has little effect on the **PV_6_Mo_6_**/Cu catalytic system in acetonitrile. Thus, we assume that in our system, the intact POM is the catalytically significant unit, and we explain the activity tendency from two vantages: (1) the redox buffer effect of **PVMo**—the difference in electrochemical properties dictates different potential buffer ranges; (2) the difference in ET rates of V and Mo in the **PVMo** Keggin complexes.

### Electrochemistry of PV_*x*_Mo_12–*x*_O_40_^(3+*x*)–^ (PVMo)

#### Effect of Water on Cyclic Voltammograms in Acetonitrile

**Figure 2:**
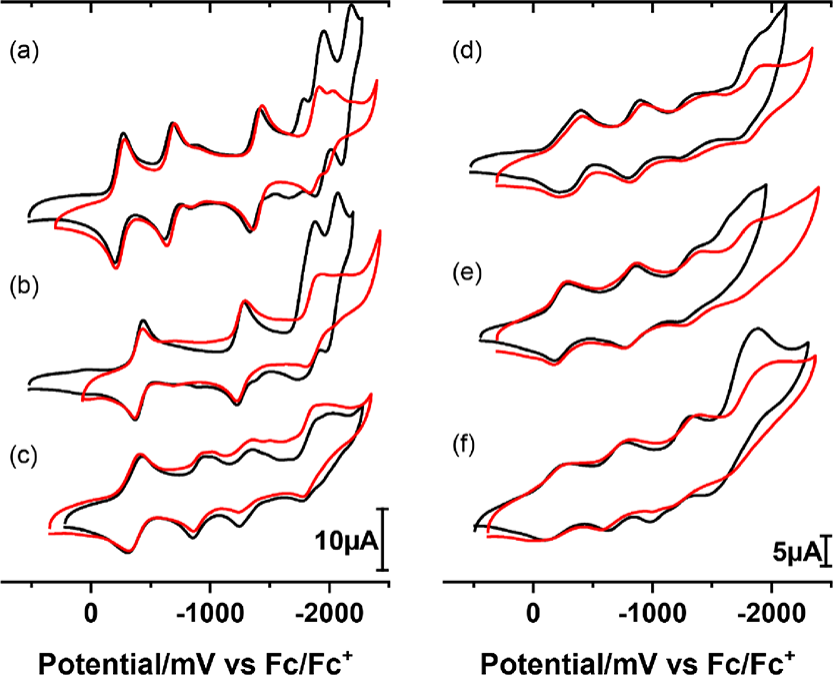
Comparison CVs in reaction acetonitrile (black) and CVs in glovebox with dry acetonitrile (red). (a) **PMo_12_**, (b) **PVMo_11_**, (c) **PV_2_Mo_10_**, (d) **PV_3_Mo_9_**, (e) **PV_4_Mo_8_**, and (f) **PV_6_Mo_6_**. 0.5 mM POM on a glassy carbon electrode. 100 mM *n*-Bu_4_NPF_6_. *v* = 100 mV s^–1^, *T* = 298 K.

In our previous work, we have found that **PV_6_Mo_6_** can accept up to six electrons in the Cu/**PVMo** catalytic system for air-based thiol oxidation.^52^ Thus, the redox properties of **PVMo** in acetonitrile at very negative potentials are important. However, trace amounts of water in acetonitrile can have a significant effect on the electrochemical behavior of reduced POMs.^63,64^ Therefore, we compared the CVs of **PVMo** in reaction acetonitrile and dry acetonitrile. Since acetonitrile can pick up small quantities of water from ambient air during laboratory manipulations, including argon purging, the dry acetonitrile CVs were conducted in the glovebox. Figure 2 compares the CVs of **PVMo** in reaction acetonitrile and dry acetonitrile. The assignment of peaks is given below.

**Figure 3:**
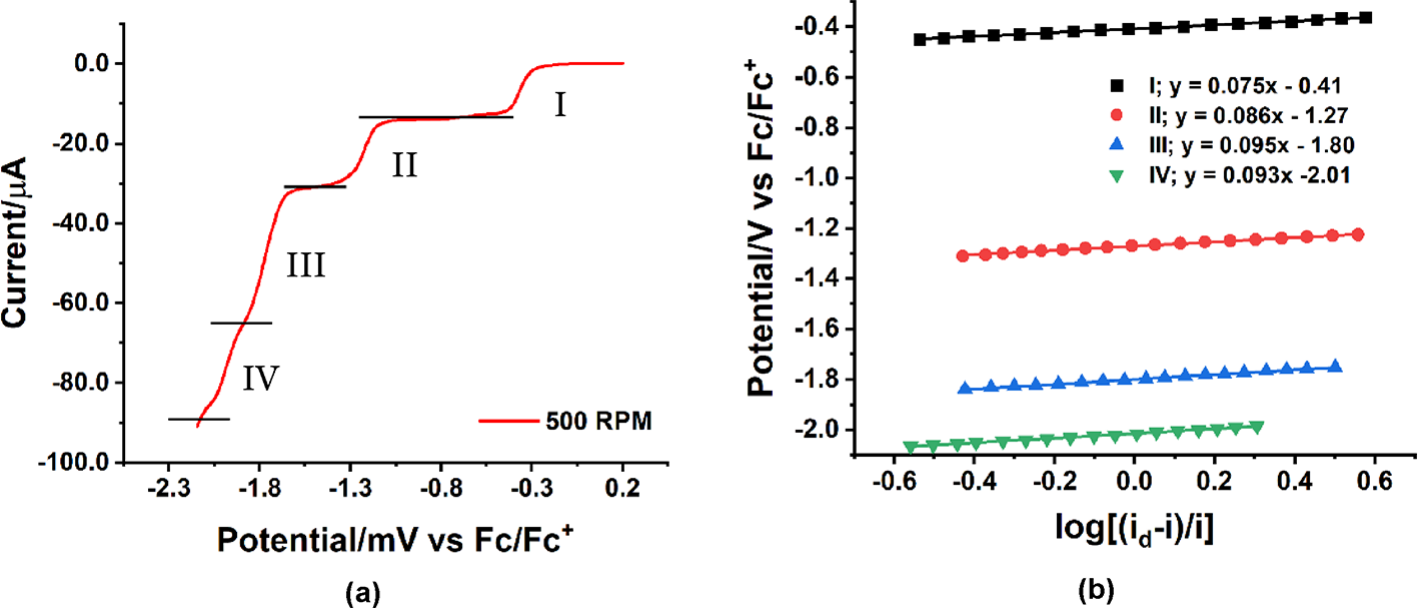
(a) Rotating disk electrode (RDE) voltammetry of **PVMo_11_** at 500 RPM. (b) *E* vs log [(*i*_d_ – *i*)/*i*] curves for four processes at the RDE. Conditions: **PVMo_11_** (0.5 mM), *n*-Bu_4_NPF_6_ (100 mM), acetonitrile (20 mL), at room temperature under argon, scan rate 5 mV s^–1^.

Figure 2a,b (black) shows that the replacement of a single molybdenum atom with vanadium (**PVMo_11_**) dramatically changes the shape of CV curves. Instead of three peaks, only two are seen for *x* = 1 in the range 0 to −1500 mV. The first peak arises from the V^V^/V^IV^ redox couple and is one-electron. This result is consistent with previous works^65,66^ and is confirmed by BE at potentials −780 and −1500 mV, each of which involve a transfer of one electron (Figure S1 and Table S1). At potentials more negative than −1500 mV, two peaks transform to two-electron ones and are very similar to those seen for **PMo_12_**. The small shoulder peak on **PMo_12_** comes from the electrochemical instability of **PMo_12_** when scanned to a very negative potential (<−1800 mV) (Figure S4). To confirm the number of electrons of the peaks at potentials negative of −1500 mV, RDE voltammetry was conducted; however, since the two peaks are quasi-reversible and too close to each other, the plateau current of the RDE scans is relatively hard to define. The approximate relative ratio of limiting currents at 500 rpm is I:II:III:IV = 1:1.3:2.3:1.9 (Figure 3a). Plots of *E* vslog[(*i*_d_ – *i*)/*i*] are linear for all four processes (Figure 3b).^67^ The slopes for the first and second confirmed one-electron processes are 75 and 86 mV, respectively, which are larger than 59 mV for a theoretical reversible one-electron process. This is consistent with relatively slow ET in acetonitrile. The third and fourth two-electron processes have slopes (93 mV) close to the one-electron process but not half of it. This is consistent with the processes III and IV being two proton-coupled one-electron processes at different potentials, but not a two-electron Nernstian process.^68^ On comparing the CVs of **PMo_12_** and **PVMo_11_** in reaction acetonitrile and dry acetonitrile, we can confirm that the two-electron transfer peaks are due to the PCET^64,69,70^ processes facilitated by the trace water present in the reaction acetonitrile, while in the glovebox samples (dry acetonitrile), the two-electron peaks change to one-electron peaks (Figure 2a,b).

The CV of **PV_2_Mo_10_** shows three peaks before −1500 mV (Figure 2c). Bulk electrolysis shows that the first peak is one-electron, while the second and third peaks share one electron with an electron ratio of 0.44:0.56 (Figure S2 and Table S2). UV–vis spectra after bulk electrolysis at each potential were collected. The spectra were compared with those from ascorbic acid titration and confirm that the second and third peaks in total constitute the transfer of one electron during bulk electrolysis (Figure S10a). The Levich plots (*i*_L_ vs ω^1/2^) from the RDE voltametric data for all four processes are linear, indicating mass transport control (Figure S5a,b). The relative ratio of limiting currents is I:II:III = 1:0.59:0.70, which confirms the bulk electrolysis results. That two peaks share one electron is attributed to the presence of two families of isomers with different free energies. This is consistent with the computational result from Neumann et al.^71^ The two-electron reduced isomers of [PV_2_Mo_10_]^7–^ with the distal vanadium centers have different free energies than the isomers with vicinal vanadium centers. According to the calculation in acetonitrile, if we assume that [PV_2_Mo_10_]^7–^ is a singlet,^72^ the average energy difference between two different groups of isomers is 13 kcal/mol = 5.4 × 10^4^ J/mol. This is roughly consistent with the data from CV that the potential difference between the second and third peaks is 386 mV = 3.7 × 10^4^ J/mol. The relative current ratio of the two peaks should be equal to the ratio of degeneracies of the two isomer families.^73^ Computational results indicate that the distribution of these positional isomers and their associated degeneracies will change with solvent;^71^ therefore, we do not discuss these features further here. Comparing the CVs of the glovebox samples reveals that the fourth peak does not obviously change and can be assigned as two one-electron processes close to each other, a result confirmed by the BE data that gives an approximate coulomb ratio of I:IV = 1:1.7.

The CVs of **PV_3_Mo_9_** and **PV_4_Mo_8_** are very similar. The first three peaks have approximately the same height, which are slightly lower than the first peak of **PVMo_11_**. They are broader not only from the presence of several **PV_3_Mo_9_** and **PV_4_Mo_8_** isomers but also because they exist as a statistical distribution.^73^ BE shows that the first three peaks of **PV_3_Mo_9_** are one-electron oxidations of vanadium (Figure S3 and Table S3), a finding that is confirmed by the relative ratio of limiting currents, I:II:III = 0.95:1:1.21. RDE voltammetry of the fourth peak does not have a well-defined plateau current due to some irreversibility (Figure S5c,d). RDE voltammetry of **PV_4_Mo_8_** shows that the first two peaks are one-electron and the relative ratio of limiting currents is I:II = 1:1.3 (Figure S5e,f). Since the third and fourth peaks of **PV_4_Mo_8_** are irreversible, BE and RDE cannot provide useful information on them. The CVs of **PV_3_Mo_9_** in dry acetonitrile (Figure 2d) shows four unequivocal one-electron peaks. Thus, the fourth peak in reaction acetonitrile can be assigned as a two-electron PCET process. The dry acetonitrile CVs of **PV_4_Mo_8_** also show four distinct one-electron peaks, while the fourth peak in reaction acetonitrile changes potential slightly and becomes less reversible. Therefore, we assign all four peaks of **PV_4_Mo_8_** as one-electron peaks in reaction acetonitrile. Finally, in the previous study, we assigned the first three peaks of **PV_6_Mo_6_** to one-electron processes while the fourth peak was assigned as a three-electron process from the RDE data. Figure 2f shows that this fourth peak of **PV_6_Mo_6_** in reaction acetonitrile is also a PCET process.

Since a trace amount of water has a large effect on the electrochemical behavior of the reduced POMs, and RSH oxidation generates water during the reaction itself, the role of added water and its role on the electrochemistry must be addressed. In these studies, the water concentration ranges from 25 to 50 mM; therefore, less than 25 mM water at a maximum is generated by the end of the reaction. Here, we use **PVMo_11_** to probe and discuss the water concentration effect. Figure S6a shows that when up to 30 mM H_2_O is added to the reaction acetonitrile, the CV peaks positive of −1.5 V do not change and the peaks negative of −1.5 V shift only slightly positive relative to those in the CVs run in the reaction acetonitrile without addition of any water. As a result, the effect of water concentration can be neglected under the conditions in this work. Figure S6b shows that by adding more H_2_O (up to 300 mM), the first vanadium peak barely changes; however, the second peak becomes less reversible. The peaks more negative than −1.5 V shift to more positive potentials as the H_2_O concentration increases; moreover, a third multi-electron peak at ca. −1.9 V appears. Finally, many studies show that in acetonitrile, on addition of an excess of a strong acid, such as trifluoromethanesulfonic acid, POMs undergo reduction by two-electron transfer PCET processes and all the peaks shift to more positive potentials.^63,66,74,75^ In this work, an excess of RSH (2-mercaptoethanol) was used to probe the oxidation reaction. Since the pK_a_ of 2-mercaptoethonal is 9.6 in water and the H^+^ dissociation is significantly less favorable in acetonitrile,^76^ RSH cannot perform as an acid to facilitate PCET processes under the conditions in this work.

Since all the reactions in this work are run in the reaction acetonitrile, we focus on the electrochemical properties of **PVMo** under these conditions and do not consider the impact of higher H_2_O and acid concentrations. We summarize the **PVMo** peak assignments in reaction acetonitrile in Table 1. We should note that, for **PVMo** (*x* > 1), the peaks more negative than −1500 mV are quasi-reversible; therefore, the number of electrons (*n*) and the corresponding redox atoms are estimated from electrochemical and titration spectral information.

**Table 1:** **PVMo** Formal Potentials (*E*_1/2_) and Number of Electrons Transferred on Corresponding Processes (*N*) Calculated from Cyclic Voltammetry in Acetonitrile

	first peak		second peak		third peak		fourth peak	
	*E*_1/2_ (mV)	*n*	*E*_1/2_ (mV)	*n*	*E*_1/2_ (mV)	*n*	*E*_1/2_ (mV)	*n*
**PMo_12_**	–236	1 (Mo)	–654	1 (Mo)	–1372	1 (Mo)	–1924	2 (Mo)
**PVMo_11_**	–405	1 (V)	–1260	1 (Mo)	–1828	2 (Mo)	–2025	2 (Mo)
**PV_2_Mo_10_**	–367	1 (V)	–916	0.4 (V)	–1302	0.6 (V)	–1854	2 (Mo)
**PV_3_Mo_9_**^a^	–318	1 (V)	–847	1 (V)	–1306	1 (V)	–1805	1 (Mo)
**PV_4_Mo_8_**^a^	–229	1 (V)	–815	1 (V)	–1300	1 (V)	–1801	1 (V)
**PV_6_Mo_6_**^b^	–202	1 (V)	–721	1 (V)	–1285	1 (V)	–1810	3 (V)

a *E*_1/2_ measured by square pulse wave voltammetry (SWV) because the quasi-reversibility of the third and fourth peaks hinders the calculation *E*_1/2_ from CV (see Figure S10).

b *E*_1/2_ measured by rotating disk electrode (RDE) voltammetry from a previous work.

#### Effect of Vanadium Centers, *x*, on the Charge of PVMo in Solution

**Figure 4:**
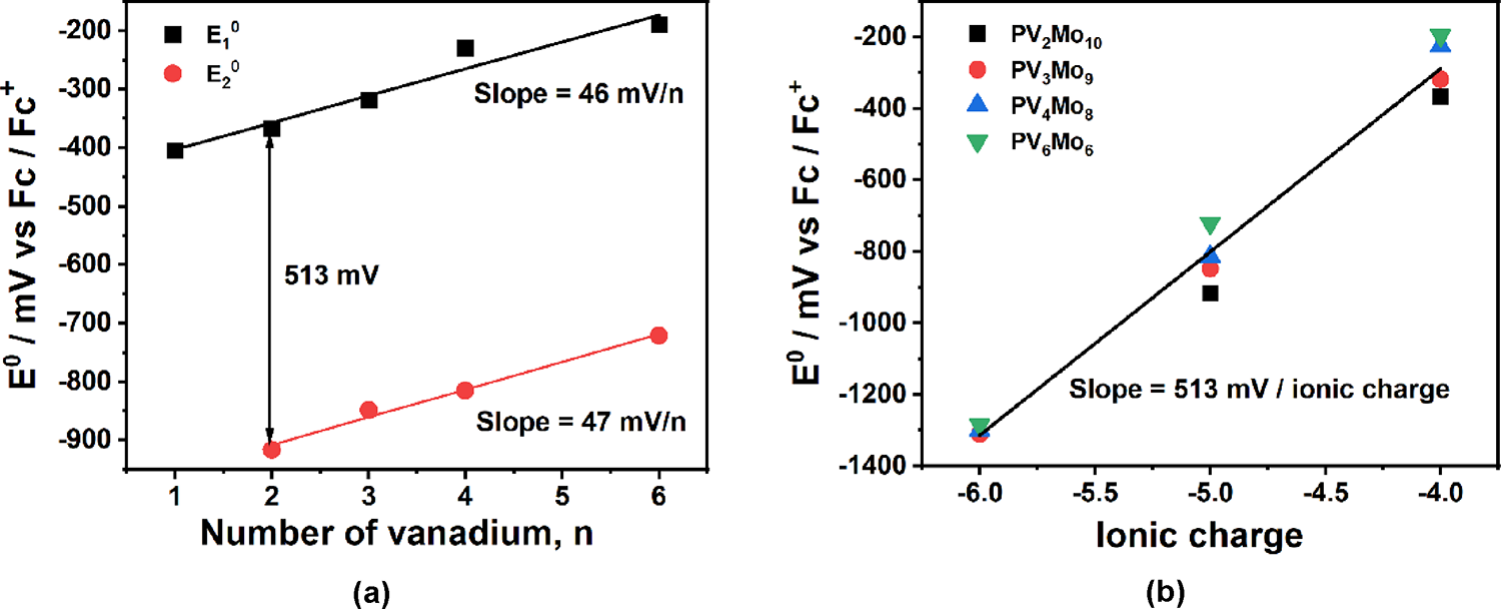
(a) First and second formal potentials (*E*_1_^0^ and *E*_2_^0^) of **PVMo** versus the number of vanadium atoms on them. (b) Formal potentials of V redox peaks (*E*^0^) versus the ionic charges on POM.

It has been reported that the first and second formal potential, *E*_1/2_, denoted as *E*_1_^0^ and *E*_2_^0^ for Keggin POMs with different heteroatoms, depends linearly on the POM charge with a slope of 430–530 mV per unit charge in aprotic solvents.^74,77,78^ In our case, the overall charge of POM depends on the number of vanadium atoms and on their reduction state. The difference in potential between the first and second vanadium redox couples for each POM is around 500 mV, which is in the same range as that for Keggin POMs with different heteroatoms (Figure 4). This indicates that the charge of POM increases by one unit upon one-electron reduction. On the contrary, the *E*_1_^0^ and *E*_2_^0^ values for all **PVMo** become more negative with the number of vanadium atoms, with a slope of only 46 mV per number of vanadium, *x* (Figure 4a). This is consistent with the interpretation that the initial charge of all studied POMs is the same but increases by one unit after accepting one electron. The number of TBA cations in all **PVMo** was confirmed by TGA in our previous work:^52^ all **PVMo** have four TBA counterions but have different numbers of protons associated with the polyanion: [PVMo_11_]^4–^, [HPV_2_Mo_10_]^4–^, [H_2_PV_3_Mo_9_]^4–^, [H_3_PV_4_Mo_8_]^4–^, and [H_5_PV_6_Mo_6_]^4–^. The electrochemistry data indicated that none of the POM protons dissociate in acetonitrile; thus, a series of **PVMo** have the same number of negative charges overall. This is further proved by the CVs as a function of POM concentration. Figure S7 shows the CVs of 0.5–2.0 mM solutions of **PV_6_Mo_6_** ([H_5_PV_6_Mo_6_]^4–^). If the **PV_6_Mo_6_** dissociates protons in acetonitrile, then intramolecular proton transfer from POM to the reduced POM ions can happen;^79^ thus, changing the POM concentration would change the acidity of the acetonitrile solution and therefore the voltametric behavior of the POMs dissolved therein. However, the potential of all the peaks remains constant for all the concentrations consistent with the series of **PVMo***not dissociating protons in acetonitrile*.

By plotting the *E*_1_^0^, *E*_2_^0^, and *E*_3_^0^ (the formal potential of the third vanadium-based voltametric peak) versus ionic charge, *i*, a line with slope of 513 mV/*i* results, which agrees very well with other studies (Figure 4b).^74,77,78^ Finally, the 46 mV/*x* potential shift from *x* = 1 to 6 can be explained by the bond valence^80−82^ difference of different **PVMo**. Eda and Osakai and Eda et al.^81,82^ correlated the bond valence of Keggin polyanion ([XW_12_O_40_]^*n*−^), with the bond length of μ_4_-O – W. We had previously correlated μ_4_-O – W and other bond lengths with Keggin polyanion isomer energies.^83^ Due to the large electronegativity of oxygen, the shorter the μ_4_-O – W bond, the greater the electron depopulation on the W atoms, inducing positive shifts in the *E*_1_^0^ potential. The properties of **PVMo** in this study depend on the length of μ_4_-O – V (*d*), since the **PVMo** have different numbers of isomers, where *d* represents an average of these bond lengths. The **PVMo** potentials shift to more positive values from *x* = 1 to 6, which corresponds to the decrease in the average value of *d* from *x* = 1 to 6.

### Reduction State Measurement and Buffer Range Determination

The apparent reduction state, *n*_app_, was defined as the average number of electrons in the reduced POM and was monitored by UV–vis. POMs can be stoichiometrically reduced by ascorbic acid or SnCl_2_ and oxidized back by Ce(IV). After adding the desired equivalents of the reducing (or oxidizing) agent, UV–vis was recorded and analyzed. The absorbances at 550 and 700 nm appeared to be linearly increasing with a number of reducing equivalents added to the system. This approach has been used for **PV_6_Mo_6_** in our previous study.^52^ The V^IV^-to-Mo^VI^ intervalence charge transfer (IVCT) band at 550 nm^69,84,85^ was used to calibrate the extinction coefficient versus the number of electrons in the reduced states of **PV_4_Mo_8_** (Figure S9). We found that ascorbic acid has limited ET ability in acetonitrile: It can only reduce the vanadium atoms but not the molybdenum atoms in **PVMo**. Therefore, SnCl_2_ was used as a reducing agent to reach higher reduction states of **PVMo**. Ce(IV) was used subsequently to reoxidize the reduced POMs to confirm the calibration at 700 nm, which results primarily from the Mo^VI^-to-Mo^V^ IVCT band.^86^ The spectra for initial reduction and subsequent reoxidation of **PVMo**, *x* ≤ 3, and the corresponding calibration curves are shown in Figures S11–13, and experimental details are given in Figure S11. A new peak around 850 nm appears after adding Ce(IV) to the SnCl_2_-reduced POM solution. Figure S14a,b shows the titration spectra using **PV_3_Mo_9_** as an example. The new peak around 850 nm very likely reflects the interaction between Ce(III) cation and the reduced POM. This was confirmed by the addition of Ce(III) salt to the reduced **PV_3_Mo_9_** solution (Figure S14c).^86^ However, the peak at 850 nm does not have a significant effect on the extinction coefficient at 700 nm (Figure S14d); thus, the calibration curve for Ce(IV) oxidative titration is also reliable.

**Figure 5:**
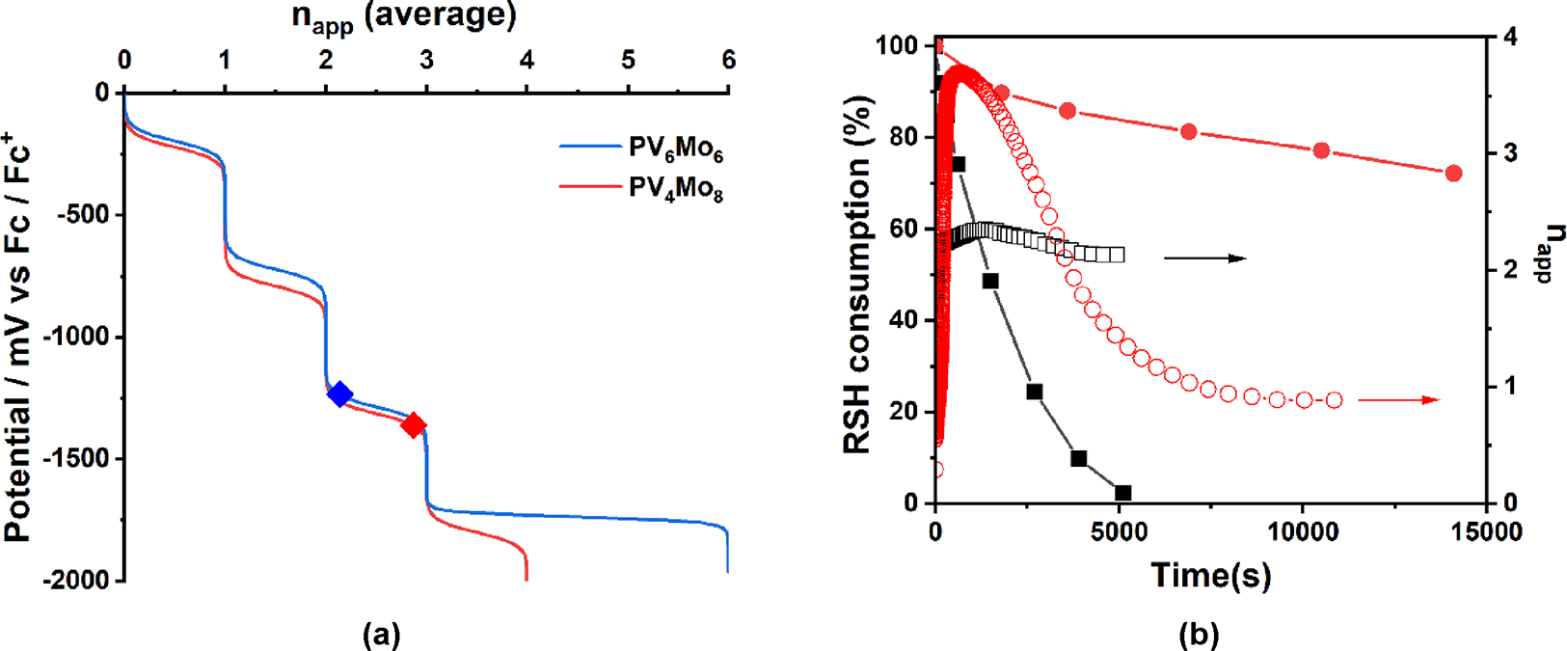
(a) The theoretical values of chemical solution potentials as a function of the average number of electrons transferred to **PVMo** (*x* = 4, 6). The diamond icons are experimental data indicating the steady reaction states. (b) RSH consumption (solid-symbol curves) and apparent reduction states, *n*_app_ (circle-symbol curves) under turnover condition: POM: 0.1 mM, Cu(II): 0.8 mM, and RSH: 30 mM. **PVMo***x* = 3, red; *x* = 6, black.

The curves of the solution potential, *E*, as a function of the reduction state, *n*_app_, define the redox buffering of this system: the number of steps, the number of electrons transferred per step, and the potential range of each step.^53,54^ The curves can be calculated according to the electrochemical information given above. The calculation and corresponding POM speciation distribution are given in Figure S15. Figure 5a shows the results for **PV_4_Mo_8_** and **PV_6_Mo_6_**, and the rest are shown in Figure S16. *n*_app_ under turnover conditions were measured experimentally; thus, the solution potential under those reaction conditions can be estimated by fitting the experimental results to the redox buffering curves. For example, under reaction conditions in Figure 1, the *n*_app_ of both **PV_4_Mo_8_** and **PV_6_Mo_6_** were measured and are shown as icons in Figure 5a.

The reduction states of all **PVMo** depend on the concentrations of RSH, Cu(II), and POM, which correspond to the change of the solution chemical potential. Figure S17e shows that under turnover conditions shown in Figure 1, the maximum *n*_app_ decreases as the number of V, *x*, on **PVMo** increases; however, unlike **PV_4_Mo_8_** and **PV_6_Mo_6_**, when *x* ≤ 3, **PVMo** cannot maintain a steady reduction state during the course of reaction. Figure 5b shows the exemplary comparison between **PV_6_Mo_6_** and **PV_3_Mo_9_**. **PV_6_Mo_6_** keeps a steady *n*_app_ around 2 to the end of conversion; however, **PV_3_Mo_9_** are quickly reduced to a maximum reduction state around 3.5 and gradually drop back to a low reduction state around 1. Figure S17 shows the *n*_app_ of all **PVMo** changes according to the Cu(II) concentration. **PV_4_Mo_8_** and **PV_6_Mo_6_** can maintain *n*_app_ from 1 to 6 (maximum *n*_app_ 4 and 6, for **PV_4_Mo_8_** and **PV_6_Mo_6_**, respectively), while **PVMo**, *x* ≤ 3, all reduced to high reduction states then dropped back to ca. 1 electron. An important feature of **PVMo**, *x* ≤ 3, is that not only V atoms but also Mo atoms are reduced in reactions where **PVMo** cannot maintain steady reduction states. The drop in *n*_app_ corresponds to the reoxidation of Mo and finally keeps a steady-state value of *n*_app_ of 1–2, which corresponds to reduced V centers only. Therefore, a key conclusion is that all vanadium centers are reduced before Mo centers and only V but not Mo can maintain a steady reduction state.

### Difference of ET Rates between V and Mo to Substrates

The overall mechanism of eq 1 was proposed in a previous work.^52^ The key step of POM as a redox buffering catalyst is the fast and reversible ET reaction in eq 2, where *n* is the number of electrons in the reduced POM.
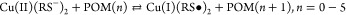
2

**Figure 6:**
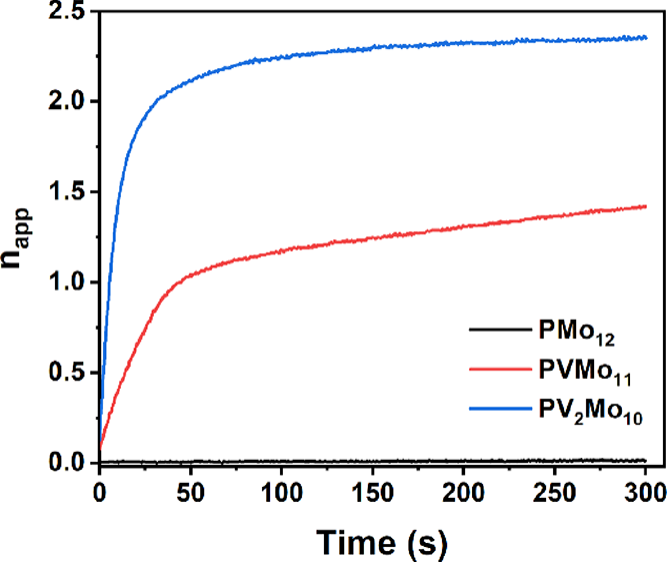
Kinetics of 0.5 mM **PMo_12_**, **PVMo_11_**, and **PV_2_Mo_10_** reduction by 50 mM RSH under Ar catalyzed by 10 μM Cu(II) in acetonitrile.

The observation that the V but not Mo atoms can keep the steady reduction state may be due to the ET rate difference between vanadium versus molybdenum to the reactant in eq 2, Cu(II)(RS^–^)_2_. Therefore, we focused on the reduction of POM by RSH catalyzed by Cu(II) under Ar. Figure 6 shows the reductions of 0.5 mM **PMo_12_**, **PVMo_11_**, and **PV_2_Mo_10_** by 50 mM RSH catalyzed by 0.1 mM Cu(II) under Ar. **PV_2_Mo_10_** accepts two electrons within 30 s, and **PVMo_11_** accepts one electron in less than 45 s, while **PMo_12_** accepts negligible electrons under these conditions. The calculated initial rates (−*d*[*n*_0_]/dt, where *n*_0_ is initial number of electrons in the reduced POM) are 0.184, 0.036, and 0.0013 s^–1^ for **PV_2_Mo_10_**, **PVMo_11_**, and **PMo_12_**, respectively. Unlike **PVMo** reduction by RSH that is catalyzed by micromolar levels of Cu(II), **PMo_12_** is only slightly reduced by RSH even at high concentrations of Cu(II) (Figure S18). The first CV peak of **PMo_12_** is more positive (*E*_1/2_ = −236 mV) than that of **PVMo_11_** (*E*_1/2_ = −405 mV; Figure 2). This suggests that the reduction rate of **PVMo_11_** and **PMo_12_** is dominated by kinetic factors. The ET rate between a substrate, Cu(II)(RS^–^)_2_, and vanadium is much faster than that between Cu(II)(RS^–^)_2_ and molybdenum in the same Keggin POM in acetonitrile. This point is further strengthened by the fact that ascorbic acid as another substrate cannot further reduce Mo atoms in **PVMo** as presented above.

**Figure 7:**
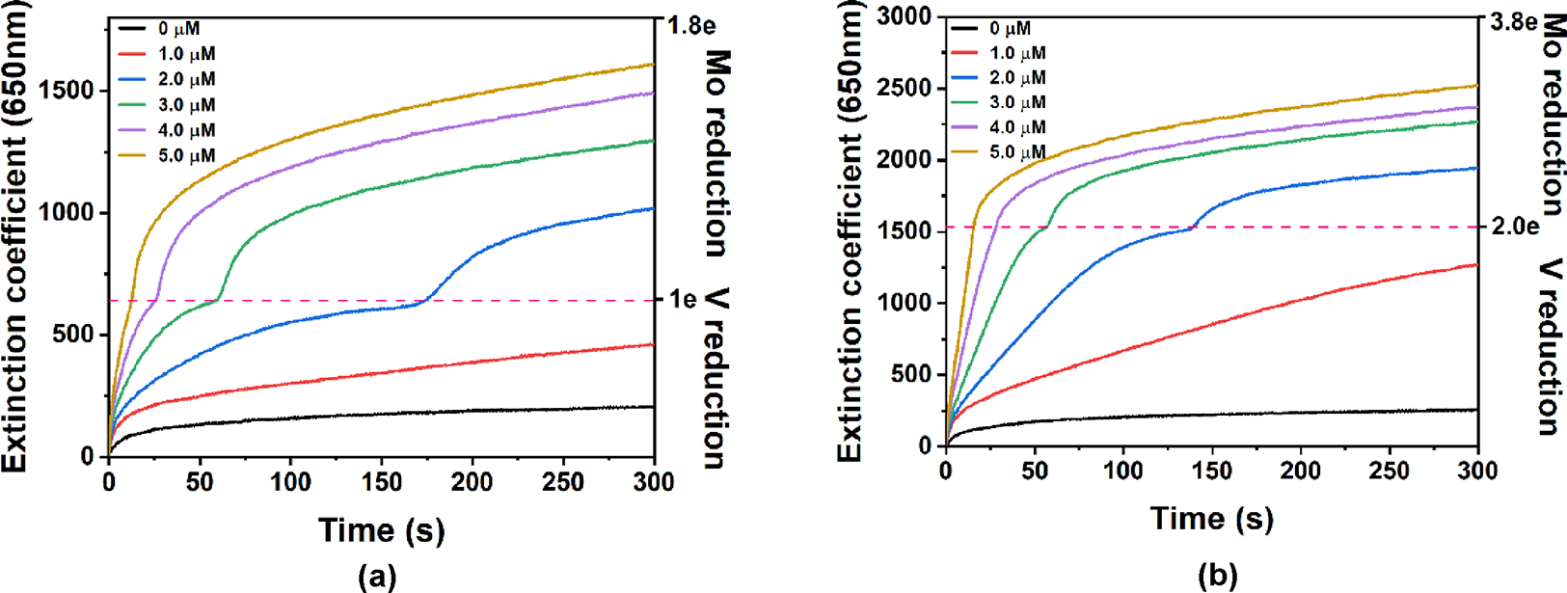
Kinetics of 0.1 mM (a) [PVMo_11_]^4–^ and (b) [PV_2_Mo_10_]^5–^ reduction by 50 mM RSH under Ar catalyzed by Cu(II) in water at pH = 2 phosphate buffer.

Control reactions for RSH reduction of Na_4_PVMo_11_O_40_, [PVMo_11_]^4–^ and Na_5_PV_2_Mo_10_O_40_, [PV_2_Mo_10_]^5–^ catalyzed by Cu(II) in pH = 2 phosphate buffer were conducted. Figure 7 shows the Cu(II) concentration dependence of [PVMo_11_]^4–^ and [PV_2_Mo_10_]^5–^ reductions. Interestingly, they show two-step kinetic curves with turning points at one- and two-electron reduction for [PVMo_11_]^4–^ and [PV_2_Mo_10_]^5–^, respectively. Bulk electrolysis at fixed potential and the corresponding UV–vis spectra (Figure S21, Tables S4 and 5) enabled the correlation of the extinction coefficient (ε) at 650 nm for the V^IV^-to-Mo^VI^ IVCT band in aqueous media^78^ with the apparent reduction state of POM, *n*_app_. Figure 7 shows that the POM reduction rate increases with increasing Cu(II) concentration; however, the point of change was fixed at one and two electrons for [PVMo_11_]^4–^ and [PV_2_Mo_10_]^5–^, respectively, independent of the Cu(II) concentration. The reduction kinetic changes with POM and RSH concentrations, but the turning point position does not depend on the concentration of either (Figure S19). This two-step kinetics suggests two important points: (1) The electrons in reduced POMs are highly localized on V atoms (one and two for [PVMo_11_]^4–^ and [PV_2_Mo_10_]^5–^, respectively), which agrees with the previous computational results.^71,78^ (2) The reduction of Mo atoms happens following the depletion of the oxidized V atoms, and the presence of the turning point indicates the ET rate difference between V and Mo centers and the Cu(II)(RSH)_2_ substrate is large. Figure S22 shows that [PMo_12_]^3–^ has a very slow reduction rate in this reaction even in the presence of high concentrations of the Cu(II) catalyst. We note here that previous work proved that the TBA salt of polyvanadotungstate (**PVW**) does not exhibit redox buffering in this reaction system. In this work, Figure S23 shows that [PVW_11_]^4–^ in aqueous buffer does not have two kinetic steps and only one V atom is reduced independent of concentrations. Therefore, redox buffering is a special property for **PVMo** and not shown by **PVW** in either acetonitrile or aqueous media.

The data in acetonitrile and aqueous buffer collectively prove that there is an ET rate difference between V and Mo atoms in Keggin vanadopolymolybdates, PV_*x*_Mo_12–*x*_O_40_^(3+*x*)–^. Since the ET rate from Mo to the substrate is slow, it cannot meet the requirement of a fast and reversible ET reaction that enables the redox buffering phenomenon. As a result, in acetonitrile, when Mo is reduced in **PVMo**, *x* ≤ 3, a constant reduction state cannot be maintained. In summary, only the V atoms in **PVMo** facilitate redox buffering; thus, the more V atoms that are present in **PVMo**, the wider the potential range in which redox buffering is possible. The multistep and multielectron redox buffering potential ranges facilitate the catalytic activity by compensating the solution electrochemical potential shift due to the consumption of the substrate.

## Conclusions

(1) The catalytic activity of PV_*x*_Mo_12–*x*_O_40_^(3+*x*)–^ (**PVMo**, *x* = 0–4 and 6) for aerobic thiol deodorization (2 RSH + ^1^/_2_ O_2_ → RSSR + H_2_O) increases with increasing number of vanadium atoms, *x*, in the polyanion. We explain this trend by both redox buffer thermodynamics and ET kinetics between V and Mo. The catalytic rates are much higher when redox buffering is a dominant effect (turnover frequency: 8 and 45 s^–1^ for **PV_3_Mo_9_** and **PV_4_Mo_8_**, respectively, Table S6).

(2) The cyclic voltammetric peaks of Keggin vanadopolymolybdates, PV_*x*_Mo_12–*x*_O_40_^(3+*x*)–^ (**PVMo**), under the above catalytic conditions reveal that the redox buffering capability derives from the number of steps, the number of electrons transferred each step, and the potential ranges of each step.

(3) The reduction of Mo centers is much slower than the reduction of V centers in the same Keggin vanadopolymolybdates under these ambient catalytic conditions: acetonitrile, thiol, ambient T, and air. As a result, the rapid multielectron redox processes that facilitate redox buffering are effective when molybdenum-based redox processes are not important. In contrast, the redox processes are effective in vanadopolymolybdates that contain three or more vanadium centers.

(4) While many O_2_-based organic substrate oxidation processes catalyzed by vanadopolymolybdates, PV_*x*_Mo_12–*x*_O_40_^(3+*x*)–^ (**PVMo**), are well-studied by other groups and some commercialized, none of these studies have noted the self-redox-buffering phenomenon. This dynamic redox self-buffering chemistry is demonstrated in our previous article,^52^ and this one might lead to faster rates for other POM-catalyzed selective oxidation of other organic compounds.

## Supporting Information Available

Experimental details, materials, and methods, including electrochemical analysis, titration methods, and redox speciation calculations (PDF)
