# Supplementary material for: Role of Multiple Vanadium Centers on Redox Buffering and Rates of Polyvanadomolybdate-Cu(II)-Catalyzed Aerobic Oxidations
Source: Inorg Chem. 2023 Mar 28;62(14):5822–30. doi: 10.1021/acs.inorgchem.3c00469 (PMC10091476; doi:10.1021/acs.inorgchem.3c00469)
Supplement: Supplementary file 1 — ic3c00469_si_001.pdf [file ic3c00469_si_001.pdf]

Supporting Information

# Role of multiple vanadium centers on redox buffering and rates of polyvanadomolybdate-Cu(II)-catalyzed aerobic oxidations

Xinlin Lu, Yurii V. Geletii*, Ting Cheng and Craig L. Hill*

*Department of Chemistry, Emory University, Atlanta, GA 30322*

**Corresponding Authors**

iguelet@emory.edu; chill@emory.edu

## Materials and Methods

All chemicals were purchased from commercial sources and used without further purification. The acetonitrile (Sigma-Aldrich HPLC grade ≥ 99.9%, ≤ 0.02% water) was used for catalytic reactions, kinetics measurements and electrochemistry outside the glovebox and is referred to subsequently as *reaction acetonitrile*). The acetonitrile from ThermoFisher (Extra Dry, ≤ 0.001%) was used for cyclic voltammograms (CVs) conducted in a MBRAUN LABmater 130 glovebox and referred to subsequently as *dry acetonitrile*. UV−vis spectra were measured with an Agilent 8453 spectrophotometer equipped with a diode array detector using a 1.0 cm optical path length quartz cuvette. $^{31}$P nuclear magnetic resonance (NMR) spectra were acquired on a Varian INOVA 400 spectrometer. The kinetics were followed using a SF-61 stopped-flow instrument (Hi-Tech Scientific, U.K.).

## Electrochemistry

Cyclic voltammograms (CVs) and bulk electrolysis (BE) data were obtained using a BAS CV-50W electrochemical analyzer. CVs were conducted in a standard three-electrode electrochemical cell with a glassy carbon disk working electrode and a platinum wire counter electrode. For experiments in acetonitrile, a $Ag/Ag^+$ (0.01 M $AgNO_3$ in $CH_3CN$) reference electrode with 0.1 M tetrabutylammonium hexafluorophosphate ($n$-$Bu_4NPF_6$) as the supporting electrolyte were used. The measured potential was converted to the $Fc/Fc^+$ scale using data measured from CV for 1.0 mM ferrocene (Fc). For experiments in aqueous buffer, Ag/AgCl was used as reference electrode with 0.1 M $KNO_3$ as supporting electrolyte. For bulk electrolysis experiments, a reticulated vitreous carbon working electrode was used as a working electrode. The working and counter electrode were separated by a porous glass sinter. The desired constant potential was applied in each

electrolysis until the current dropped to <10% of the initial value, then aliquots were withdrawn, and the UV-Vis spectra were recorded under Ar. The electrolysis was then resumed at the more negative potential as listed in Table S1-S5.

Rotating disk electrode (RDE) voltammetry and square pulse wave voltammetry (SWV) were conducted on a Wavedriver 10 potentiostat/galvanostat (Pine Research Instrumentation). For both experiments, the standard three electrode setup was used with a 3-mm diameter glassy carbon disk working electrode, a $Ag/Ag^+$ (0.01 M $AgNO_3$ in $CH_3CN$) reference electrode, and a platinum wire counter electrode. The rotation speed from 500-3000 RPM was controlled by a Model AFMSRCE ring-disk electrode system (Pine Research Instrumentation).

**Synthesis of TBA salts of $PV_xMo_{12-x}O_{40}^{(3+x)-}$ (x = 0-4 and 6)**

$H_3PMo_{12}O_{40}$, $H_4PVMo_{11}O_{40}$, $H_5PV_2Mo_{10}O_{40}$, $H_6PV_3Mo_9O_{40}$, $(NH_4)_7PV_4Mo_8O_{40}$ and $(NH_4)_5H_4PMo_6V_6O_{40}$ were synthesized according to the literature methods.[1–4] The tetra-*n*-butylammonium (TBA) salts of POMs were precipitated with TBA bromide from aqueous solutions with different pH values. Specifically, 0.1 M $HClO_4$ for $TBA_3PMo_{12}O_{40}$ (**$PMo_{12}$**); 0.1 M sulfate buffer at pH 2.0 for $TBA_4PVMo_{11}O_{40}$ (**$PVMo_{11}$**) and $TBA_4HPV_2Mo_{10}O_{40}$ (**$PV_2Mo_{10}$**); DI water for $TBA_4H_2PV_3Mo_9O_{40}$ (**$PV_3Mo_9$**), $TBA_4H_3PV_4Mo_8O_{40}$ (**$PV_4Mo_8$**) and $TBA_4H_5PMo_6V_6O_{40}$ (**$PV_6Mo_6$**). All the TBA salts of the POMs were recrystallized twice in acetonitrile. All the TBA salts of **PVMo** were characterized by UV-vis, ATR FT-IR and thermogravimetric (TGA) as in in previous work.[5] Here $^{31}P$ NMR spectra of **PVMo** in acetonitrile-d3 with respect to 85% $H_3PO_4$ (0 ppm) were given in Figure S24.

For $^{31}P$ NMR spectra, **$PVMo_{11}$** has a single peak at -4.31 ppm that proves its purity. **$PV_2Mo_{10}$** has a peak at -4.31 ppm indicating the **$PVMo_{11}$** component and a broad peak that split to -4.54 and -4.60 ppm which is assigned to **$PV_2Mo_{10}$** and **$PV_3Mo_9$** components. For **$PV_3Mo_9$**, in addition to the peaks that have essentially the same chemical shifts as for **$PV_2Mo_{10}$**, multiple peaks more positive than -4ppm may be assigned to **$PV_4Mo_8$** components. **$PV_4Mo_8$** and **$PV_6Mo_6$** all show multiple peaks that cannot be clearly assigned indicating the many components and positional isomers present. It is well-establishd that heterpolyacids, $H_{3+x}PV_xMo_{12-x}O_{40}^{(3+x)-}$, when x>1, are mixtures of positional isomers and components with different x.[6] The $^{31}P$ NMR data in this work shows that the TBA salts of **PVMo** in acetonitrile are isomeric mixtures.

**RSH oxidation and measurement of the varying PVMo reduction states**

2-Mercaptoethanol was used as an exemplary substrate for probing the aerobic thiol oxidation, eq 1 in the text, where RSH is 2-mercaptoethanol. The mechanism of the **$PV_6Mo_6$**/Cu system was

thoroughly studied in previous work.[5] This article focuses on the impact of the number of vanadium atoms (x = 0-4, and 6) in $PV_xMo_{12-x}O_{40}^{(3+x)-}$ (**PVMo**). The RSH concentration was quantified using Ellan's reagent (5,5-dithiobis(2-nitrobenzoic acid) (DTNB)).[7] In a typical reaction, 0.1 mL of DTNB solution (5 mg/mL in methanol) was added to a 5 mL pH = 7.4 phosphate buffer solution (50 mM). This solution was first used as the blank for UV-vis measurements. Then, a 10 μL aliquot of the reaction solution was added and the absorbance at 412 nm was followed and the RSH concentration calculated.

In a typical RSH oxidation reaction, POM (0.1 mM), $Cu(ClO_4)_2$ (0.8 mM) and 2-mercaptoethanol (30 mM) were stirred in acetonitrile in a heavy-wall glass pressure vessel in an air-conditioned room at 25±2 °C. Aliquots of the solution were withdrawn every several minutes and monitored by UV-vis spectra as described above.

In a typical **PVMo** reduction state measurement, **PVMo** (0.1 mM) and $Cu(ClO_4)_2$ (0.5 mM) were stirred in acetonitrile in a 1.0 cm optical path length quartz cuvette purged with air at 25±2 °C. After adding the 2-mercaptoethanol (30 mM), the UV-vis spectra of the solution in the course of the reaction were monitored. The absorption was then converted to the apparent extinction coefficient using the Beer–Lambert law. The average number of electrons transferred to the POM was calculated from the calibration curve using different titration methods (Figures S9-14). For experiments in acetonitrile, ascorbic acid and Ce(IV) were used for titration of **$PV_4Mo_8$** and **$PV_6Mo_6$**; $SnCl_2$ and Ce(IV) were used for titration of **$PVMo_{11}$**, **$PV_2Mo_{10}$** and **$PV_3Mo_9$**. Bulk electrolysis titration was used to confirm certain results. For experiments in aqueous condition, bulk electrolysis titration was performed for $[PVMo_{11}]^{4-}$, $[PV_2Mo_{10}]^{5-}$ and $[PVW_{11}]^{5-}$ (Figure S21).

**Stopped-Flow Measurements**

A stopped-flow UV-vis spectrometer was used to monitor the rates of $PV_nMo_{12-n}O_{40}^{(3+n)-}$ reduction by 2-mercaptoethanol at different concentrations of $Cu(ClO_4)_2$ under argon. In a typical measurement, one feeding syringe was filled with the de-aerated stock acetonitrile or aqueous buffer solution of POM and $Cu(ClO_4)_2$. The second feeding syringe was filled with the de-aerated acetonitrile or aqueous buffer solution of 2-mercaptoethanol. In all stopped-flow kinetic measurements, the concentrations of all components, POM, $Cu(ClO_4)_2$ and 2-mercaptoethanol in the reaction were two times lower than concentrations in the feeding syringes.

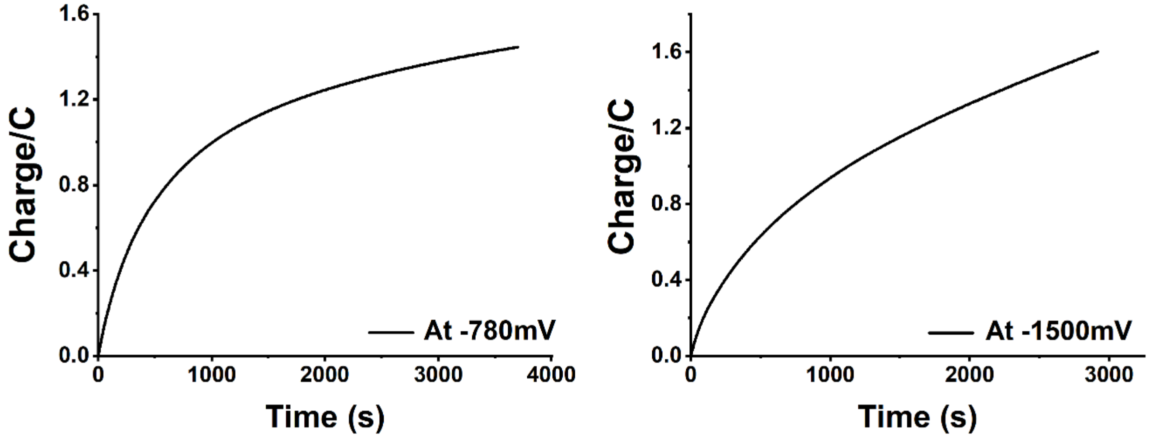


**Figure S1.** Charge vs time curves from the bulk electrolysis of **PVMo$_{11}$**. Conditions: **PVMo$_{11}$** (0.5 mM), *n*-$Bu_4NPF_6$ (100 mM) in acetonitrile (30 mL), at 25 ± 2 °C under argon. See Table S1 below.

**Table S1.** The results of bulk electrolysis of **PVMo$_{11}$** at constant potential in acetonitrile.[a]

| Potential/mV vs Fc/Fc$^+$ | Number of Coulombs | Number of electrons[b] | Ending current ratio[c]/% |
|---|---|---|---|
| -780 | 1.48 | 1.0 | 3 |
| -1500 | 1.6 | 1.1 | 5.5 |

[a] Conditions: **PVMo$_{11}$** (0.5 mM), *n*-$Bu_4NPF_6$ (100 mM), acetonitrile (30 mL), at room temperature under argon. [b] Number of electrons transferred to the POM (polyanion) unit, calculated according to Faraday's law of electrolysis. [c] Ending current ratio is defined as the final current at the end of the bulk electrolysis over the initial current at the beginning of the bulk electrolysis at the specific potential.

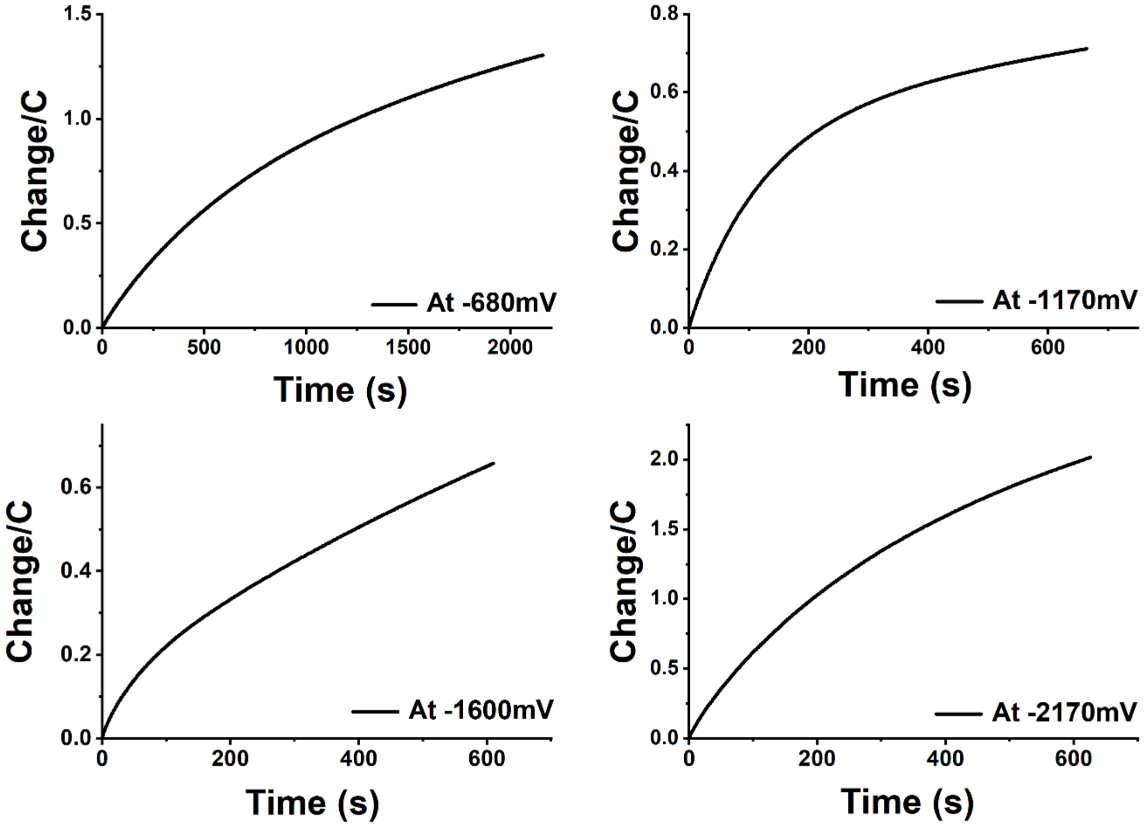


**Figure S2.** Charge vs time curves from the bulk electrolysis of **$PV_2Mo_{10}$**. Conditions: **$PV_2Mo_{10}$** (0.5 mM), *n*-$Bu_4NPF_6$ (100 mM) in acetonitrile (30 mL), at 25 ± 2 °C under argon. See Table S2 below.

**Table S2:** The results of bulk electrolysis of **$PV_2Mo_{10}$** at constant potential in acetonitrile.[a]

| Potential/mV vs $Fc/Fc^+$ | Number of Coulombs | Number of electrons | Ending current ratio/% |
|---|---|---|---|
| -680 | 1.3 | 0.9 | 8.2 |
| -1170 | 0.6 | 0.4 | 3.7 |
| -1600 | 0.75 | 0.5 | 8.9 |
| -2170 | 2.2 | 1.6 | 11 |

[a] Conditions: **$PV_2Mo_{10}$** (0.5 mM), *n*-$Bu_4NPF_6$ (100 mM), acetonitrile (30 mL), at room temperature under argon.

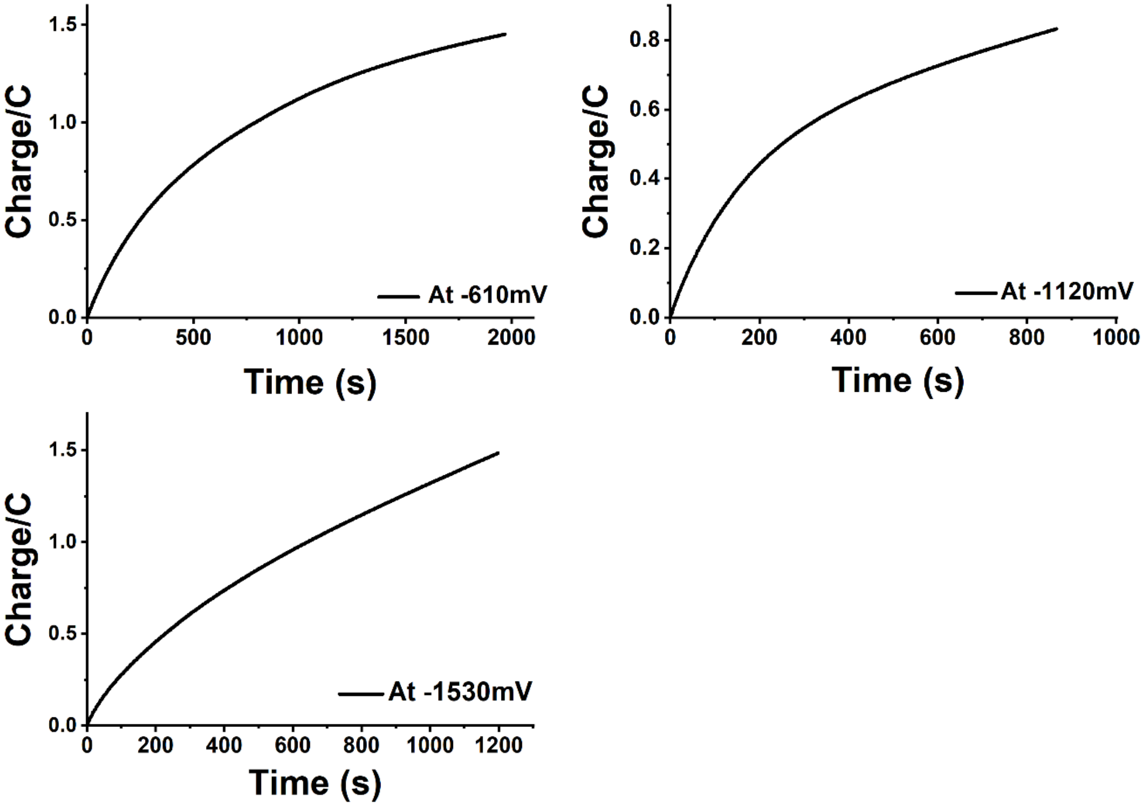


**Figure S3.** Charge vs time curves from the bulk electrolysis of **$PV_3Mo_9$**. Conditions: **$PV_3Mo_9$** (0.5 mM), *n*-$Bu_4NPF_6$ (100 mM) in acetonitrile (30 mL), at 25 ± 2 °C under argon. See Table S3 below.

**Table S3:** The results of bulk electrolysis of **$PV_3Mo_9$** at constant potential in acetonitrile.[a]

| Potential/mV vs $Fc/Fc^+$ | Number of Coulombs | Number of electrons | Ending current ratio/% |
|---|---|---|---|
| -610 | 1.45 | 1.0 | 5.2 |
| -1120 | 0.82 | 0.6 | 5.5 |
| -1530 | 1.4 | 1.0 | 10 |

[a] Conditions: **$PV_3Mo_9$** (0.5 mM), *n*-$Bu_4NPF_6$ (100 mM), acetonitrile (30 mL), at room temperature under argon.

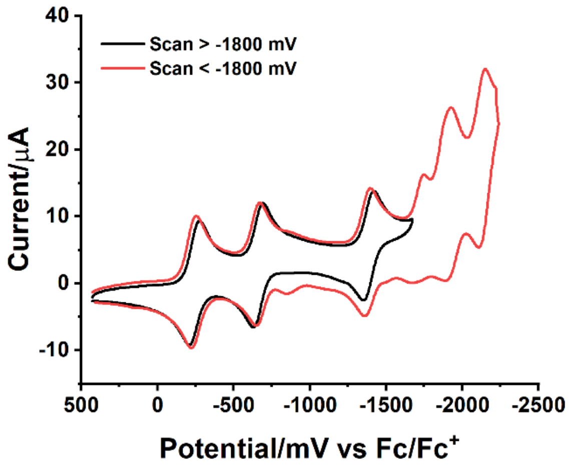


**Figure S4.** Electrochemical instability of **PMo$_{12}$** when scanned negative of -1800 mV.

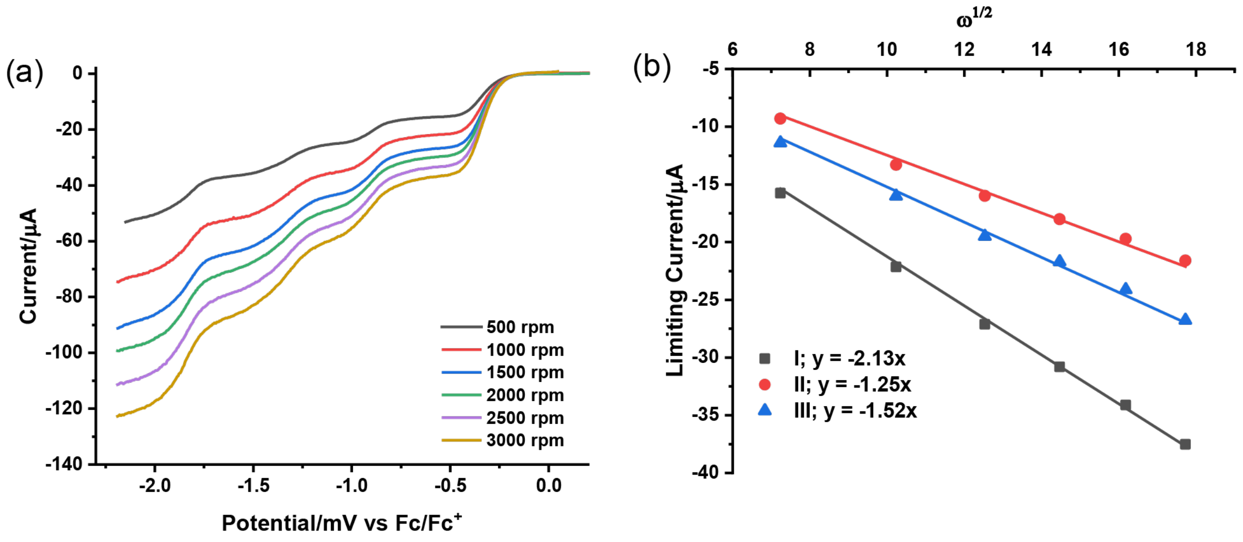

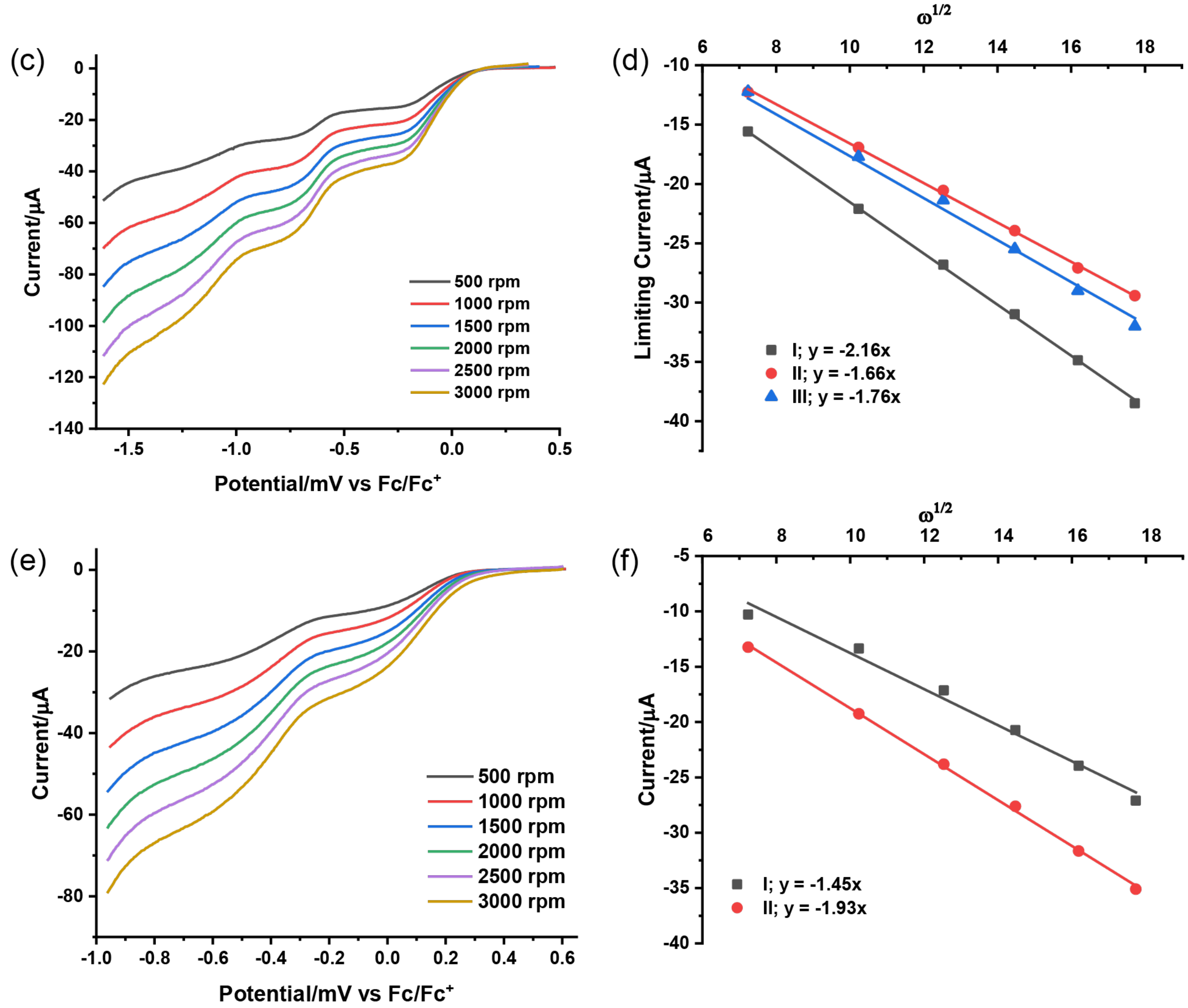


**Figure S5.** Rotating disk electrode (RDE) voltametric data and the corresponding Levich plots (limiting current, $i_L$ vs $\omega^{1/2}$). Conditions: POM (0.5 mM), *n*-$Bu_4NPF_6$ (100 mM), acetonitrile (20 mL), at room temperature under argon, scan rate 5 mV $s^{-1}$. (a, b) **$PV_2Mo_{10}$**; (c, d) **$PV_3Mo_9$**; (e, f) **$PV_4Mo_8$**.

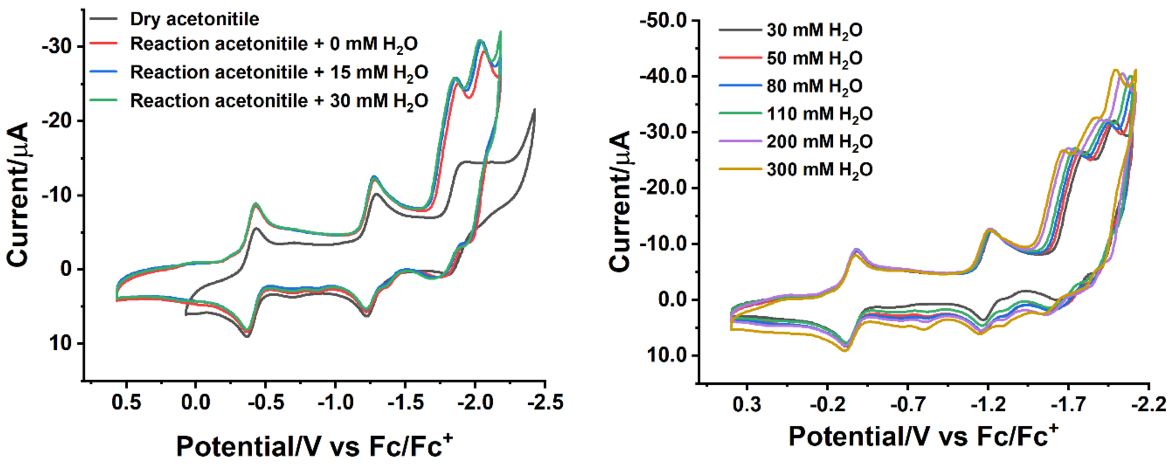


**Figure S6.** CVs of **$PVMo_{11}$** in different concentrations of water. (a) CVs in dry acetonitrile, reaction acetonitrile and reaction acetonitrile with small amount of water that the RSH oxidation reaction generates. (b) CVs with larger quantities of added water.

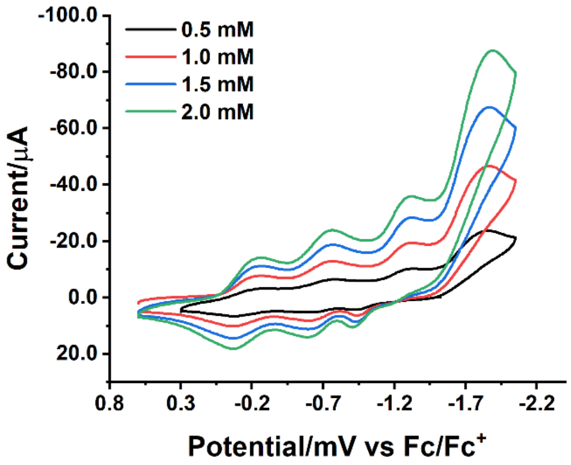


**Figure S7.** The effect of analyte (**$PV_6Mo_6$**) concentration on the CV behavior.

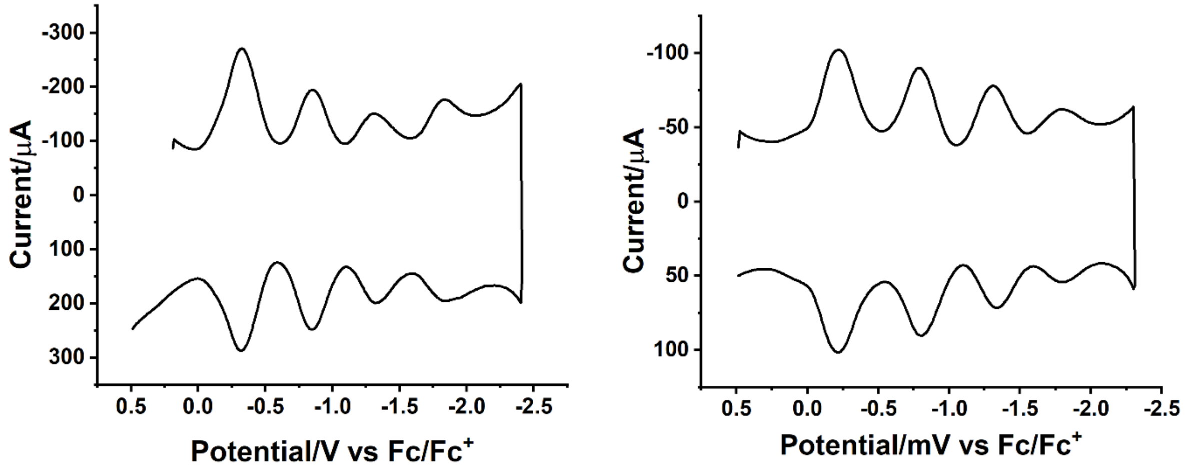


(a) (b)

**Figure S8.** Square pulse wave voltammetry (SWV) of (a) **$PV_3Mo_9$** and (b) **$PV_4Mo_8$**. Conditions: POM (0.42 mM), *n*-$Bu_4NPF_6$ (250 mM), amplitude 100 mV, period 10 ms, increment 10 mV and sampling width 1 ms.

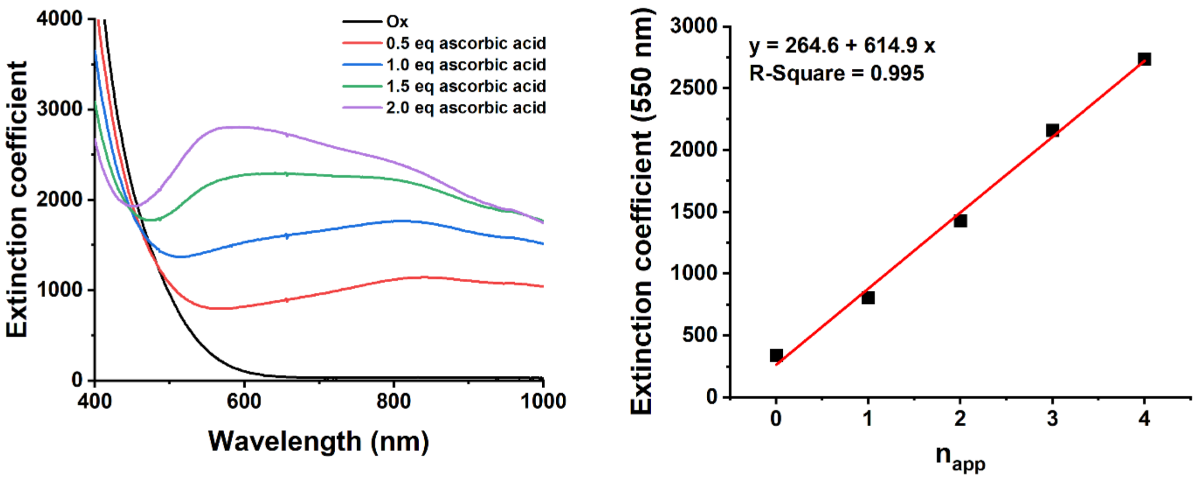


(a) (b)

**Figure S9.** (a) Ascorbic acid titration of **$PV_4Mo_8$** followed by UV-Vis spectra. For each data point, 0.5 equivalent of ascorbic acid, a two-electron reductant under these conditions, is added. (b) Calibration curve: extinction coefficient at 550 nm versus the number of electrons transferred by ascorbic acid, $n_{app}$.

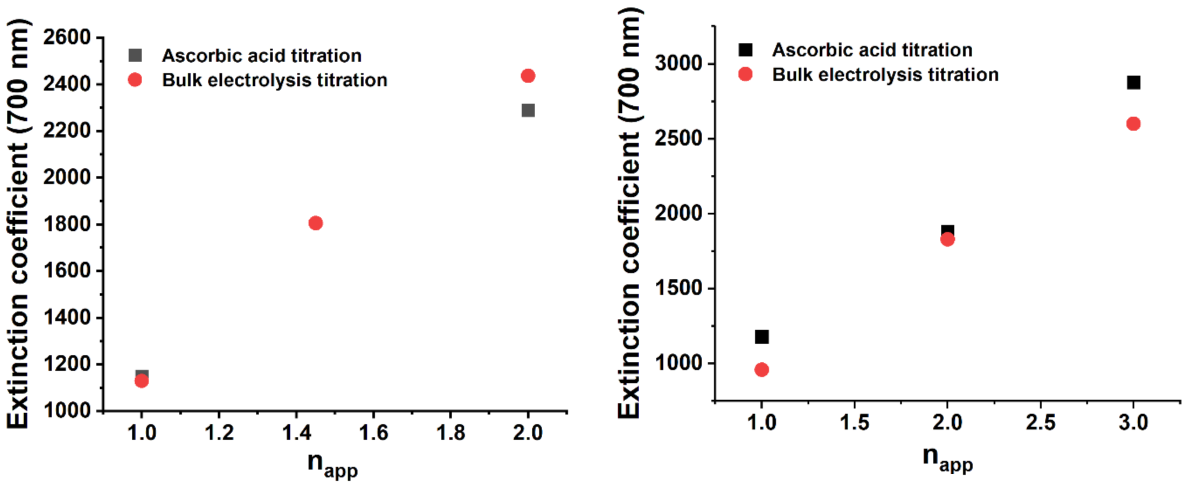


(a) (b)

**Figure S10** Calibrations by ascorbic acid and bulk electrolysis titration: the extinction coefficient at 700 nm versus number of electrons transferred, $n_{app}$. (a) **$PV_2Mo_{10}$**; (b) **$PV_3Mo_9$**.

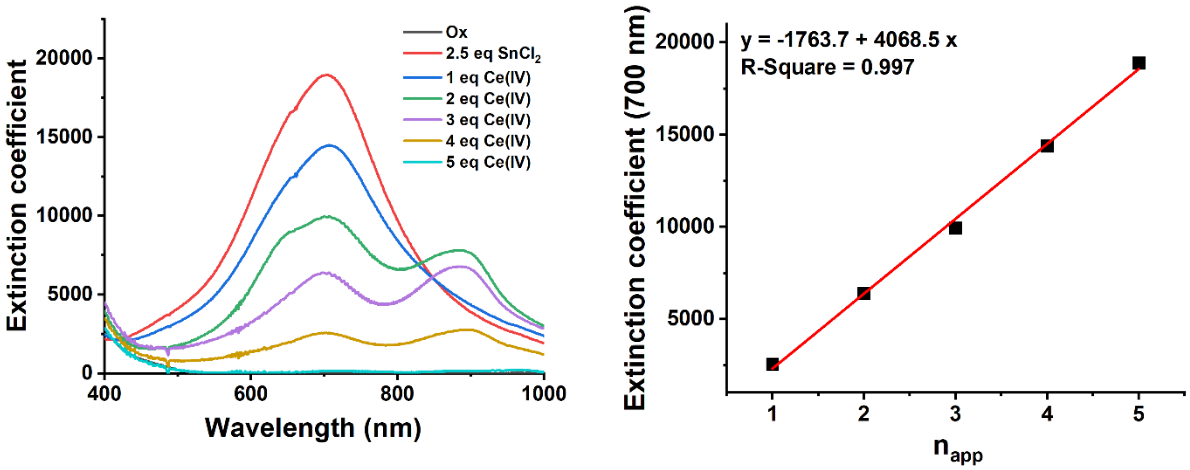


(a) (b)

**Figure S11.** (a) Reductive ($SnCl_2$) and subsequent oxidative Ce(IV) titrations of **$PVMo_{11}$** monitored by UV-Vis spectra. POM (0.1 mM) was reduced by 2.5 equivalents of $SnCl_2$ by direct addition of solid $SnCl_2$, followed by oxidative titration via addition of 1 equivalent Ce(IV). Each UV-Vis spectrum was recorded when the reduced POM was fully reoxidized back to its initial state (Ox). The Ce(IV) stock solution was prepared by dissolving ammonium cerium (IV) nitrate (Ce(IV)) in 0.1 M aqueous $HClO_4$ and using 50 µL stock solution per equivalent of reduced **$PVMo_{11}$**. (b) Linear calibration curve of extinction coefficient versus number of electrons transferred to the polyanion, $n_{app}$, evaluated at 700 nm.

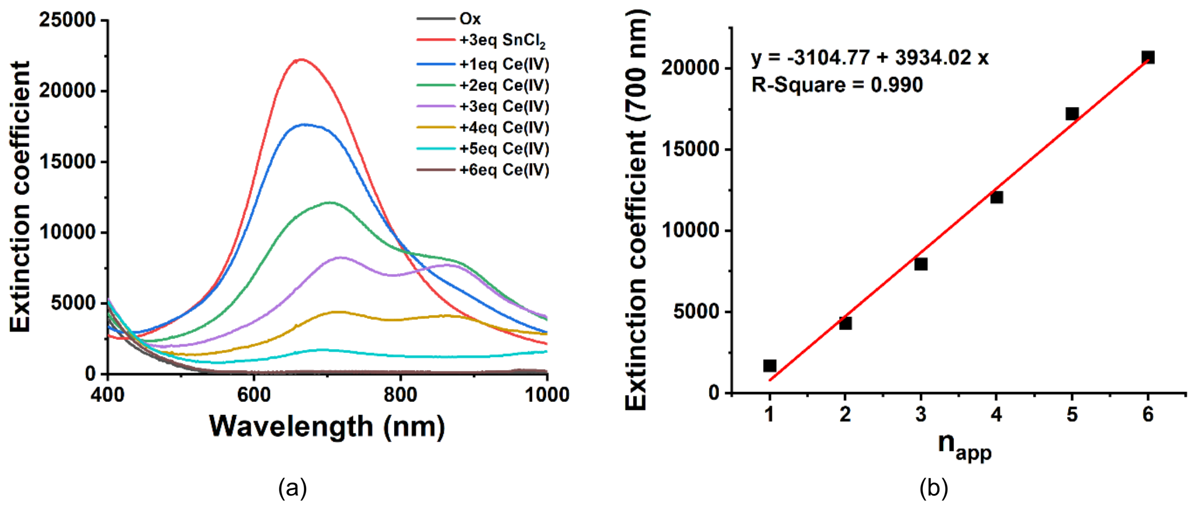


**Figure S12.** (a) Reductive ($SnCl_2$) and subsequent oxidative Ce(IV) titrations of **$PV_2Mo_{10}$**. (b) Linear calibration curve of extinction coefficient versus number of electrons transferred to the polyanion, $n_{app}$, evaluated at 700 nm.

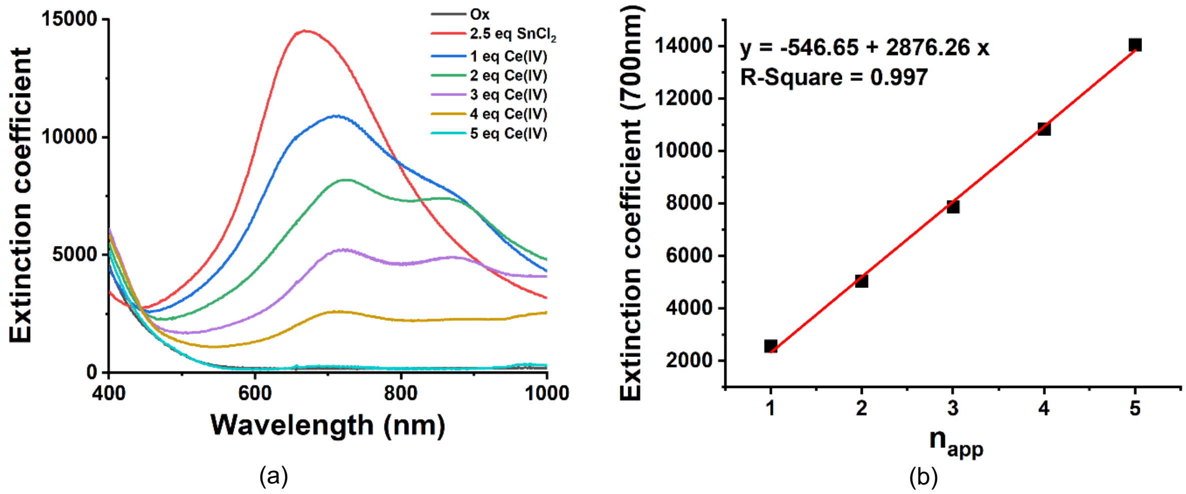


**Figure S13.** (a) Reductive ($SnCl_2$) and subsequent oxidative Ce(IV) titrations of **$PV_3Mo_9$**. (b) Linear calibration curve of extinction coefficient versus number of electrons transferred to the polyanion, $n_{app}$, evaluated at 700 nm.

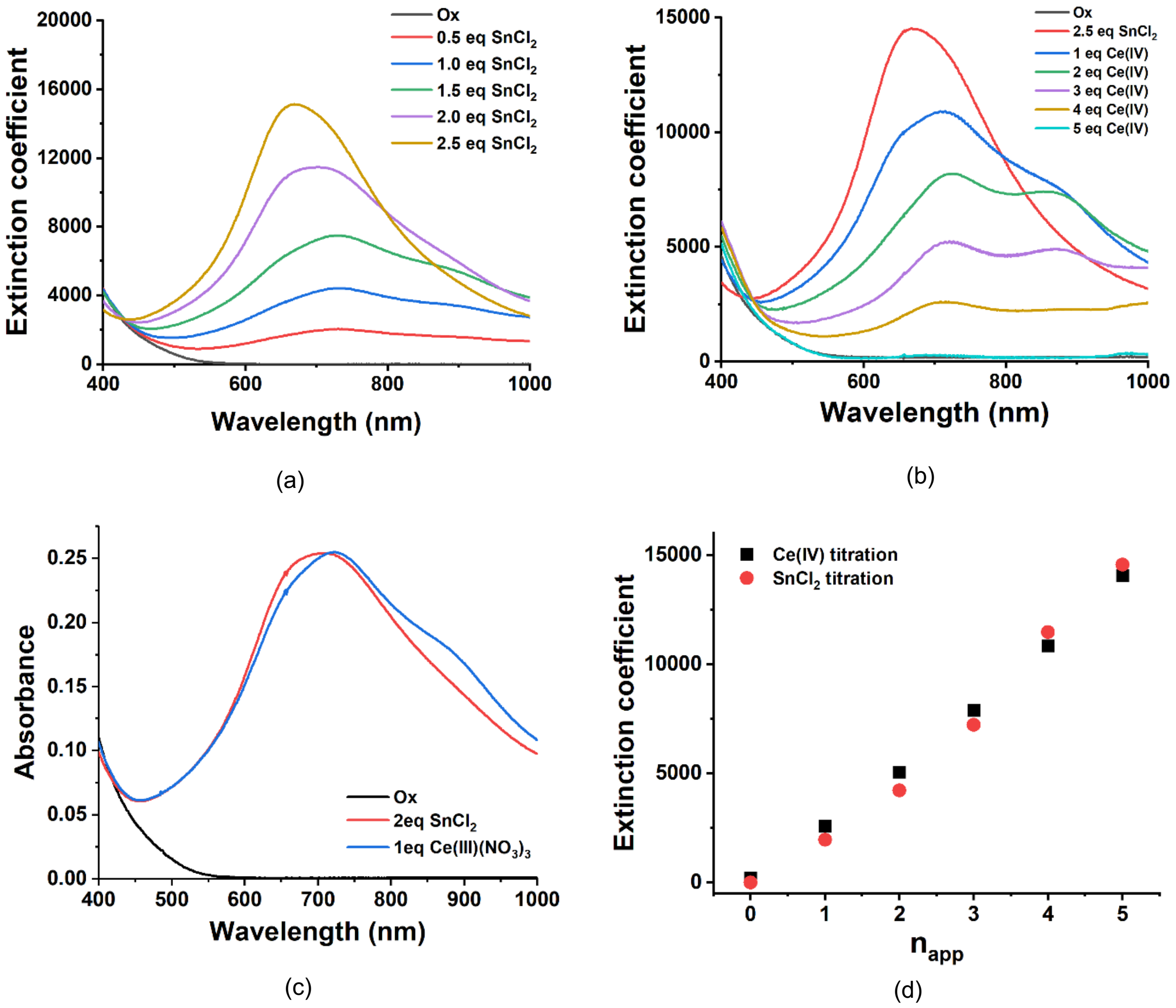


**Figure S14.** (a) **$PV_3Mo_9$** reductive titration by $SnCl_2$. (b) Five-electron-reduced **$PV_3Mo_9$** from addition of 2.5 equivalent of $SnCl_2$ then oxidative titration by Ce(IV). (c) Comparison of spectra before and after adding $Ce(III)(NO_3)_3$ to four-electron-reduced **$PV_3Mo_9$**. (d) Calibration curves at 700 nm for $SnCl_2$ reductive titration followed by Ce(IV) oxidative titration of **$PV_3Mo_9$**.

## Quantifying the speciation of reduced POMs

Here we use **PVMo₁₁** as an example to calculate the POM distribution in different reduction states. However, the same procedure was used for the other **PVMo**. This distribution depends on the chemical solution potential, E, as described by eq S1, where $E_i$ is the standard reduction potential of a $(\mathbf{PVMo_{11}})_i$ / $(\mathbf{PVMo_{11}})_{i+1}$ couple measured electrochemically

$$\alpha_i = (\alpha_{i-1}10^{(Ei - E)/60})/(\textstyle\sum_{i=0}^{i=6} \alpha i) \qquad \text{(S1)}$$

The calculated apparent reduction state of POM, $n_{app}$ (average), is given in eq S2, and the results are given in Figure S15.

$$n_{app}\text{ (average)} = \textstyle\sum_0^6 i(\alpha i) \qquad \text{(S2)}$$

The third and fourth peaks of **$PVMo_{11}$** are two-electron (see Figure 1). For reversible two-electron peak in the CV, $\Delta E\ (E_4 - E_3 \text{ and } E_6 - E_5) > 0$.[8] Thus, we assume that $E_4 = E_3 = -1828$ mV and $E_6 = E_5 = -2025$ mV.

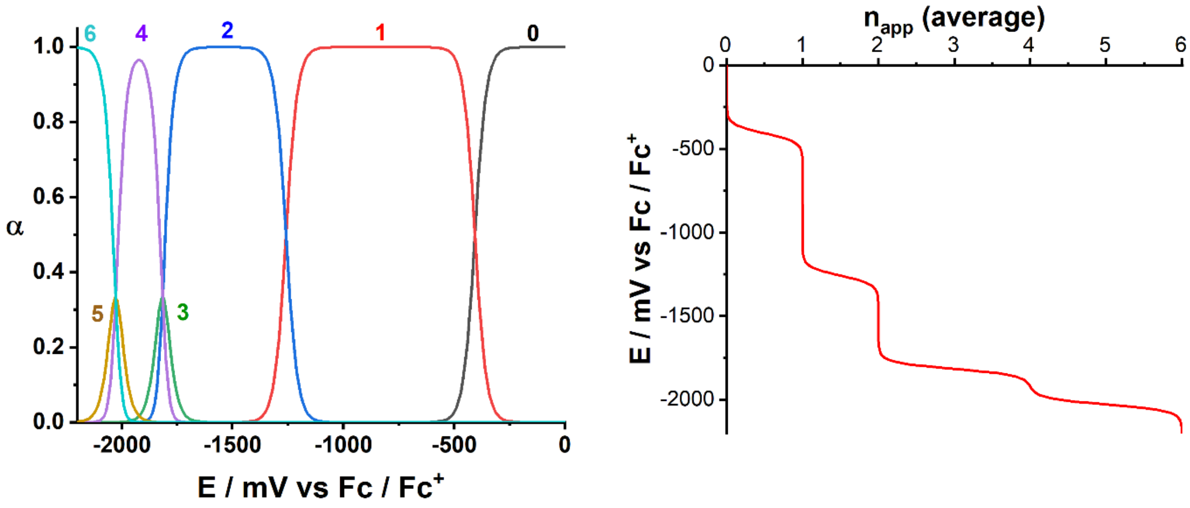


(a) (b)

**Figure S15.** (a) The distribution diagram of the reduced forms of **$PVMo_{11}$** as a function of chemical solution potential, E. (b) The theoretical values of chemical solution potentials as function of the reduction state, $n_{app}$, of **$PVMo_{11}$**.

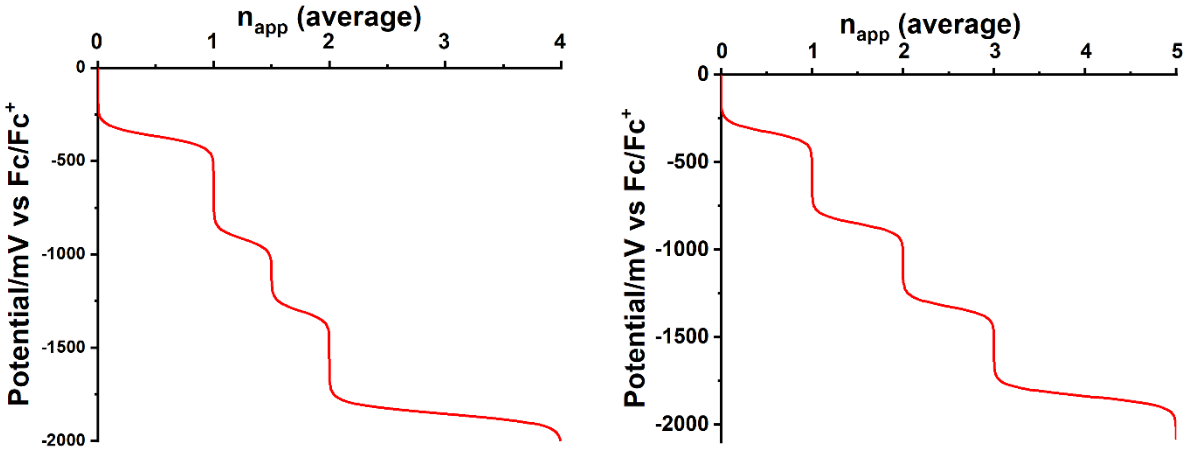


(a) (b)

**Figure S16** The theoretical values of chemical solution potentials as function of the reduction state, $n_{app}$. (a) **$PV_2Mo_{10}$**; (b) **$PV_3Mo_9$**.

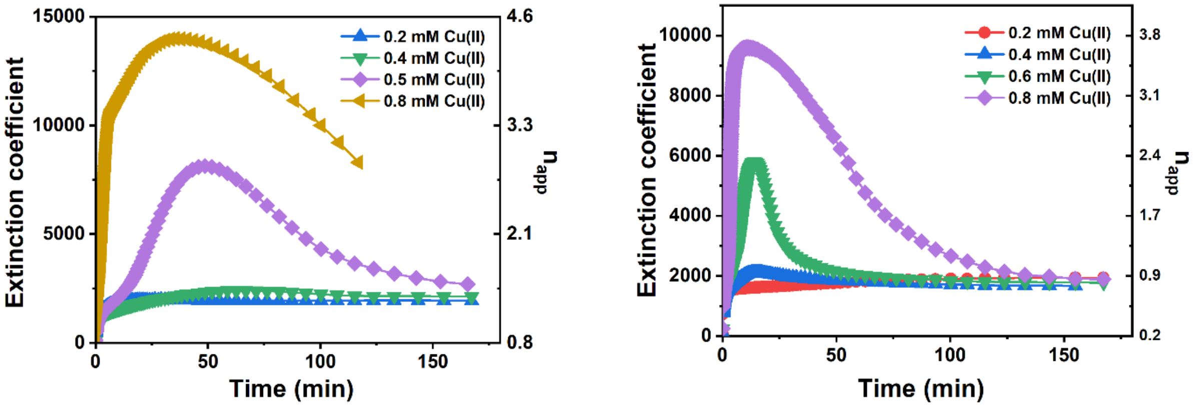


(a) (b)

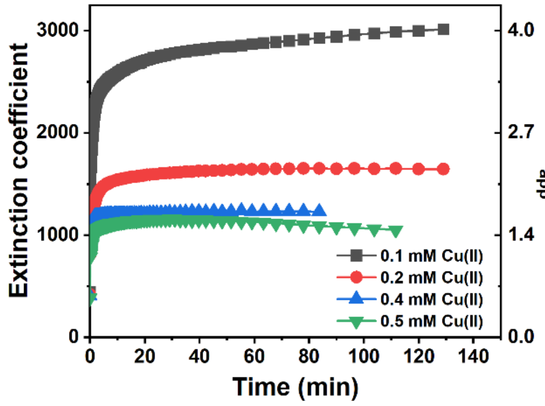


(c)

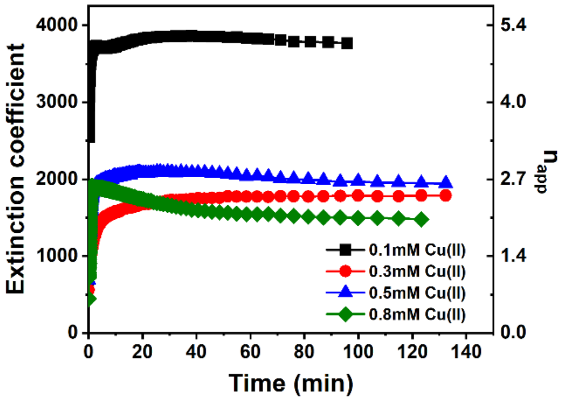


(d)

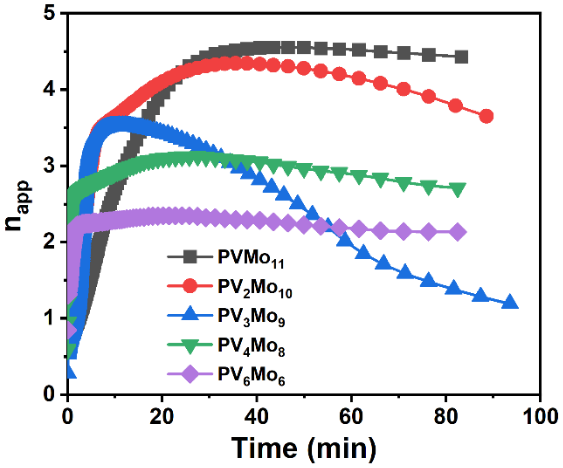


(e)

**Figure S17.** $n_{app}$ of **PVMo** as function of Cu(II) concentration with 30 mM RSH and 0.1 mM POM. (a) **$PV_2Mo_{10}$**; (b) **$PV_3Mo_9$**;(c) **$PV_4Mo_8$**; (d) **$PV_6Mo_6$**; (e) $n_{app}$ comparison of **PVMo,** 30 mM RSH and 0.1 mM POM and 0.8 mM Cu(II).

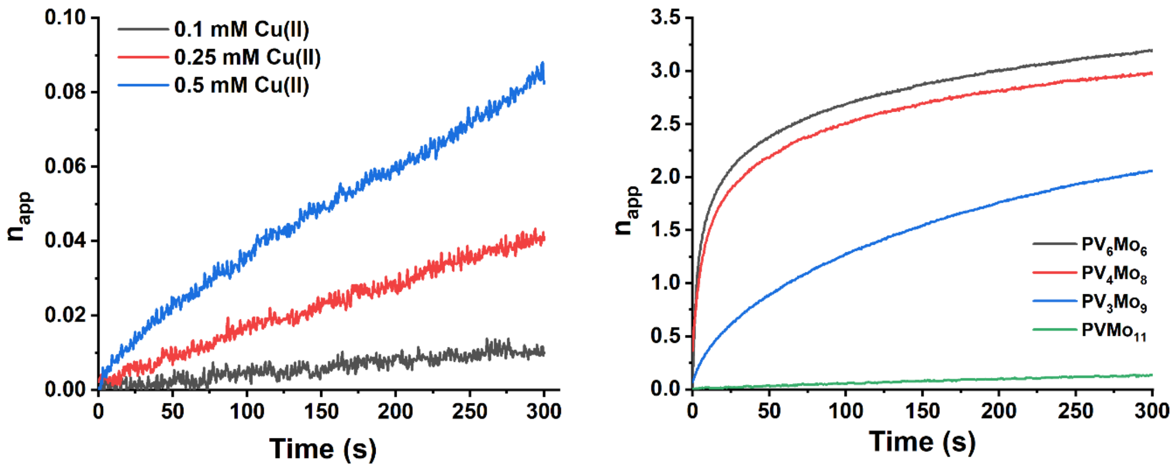


(a) (b)

**Figure S18.** (a) Kinetics of 0.5 mM **$PMo_{12}$** reduction by 50 mM RSH: dependence on Cu(II) concentration under Ar. (b) Kinetics of 0.5 mM **PVMo** reduction by 25 mM RSH catalyzed by 2μM Cu(II) under Ar.

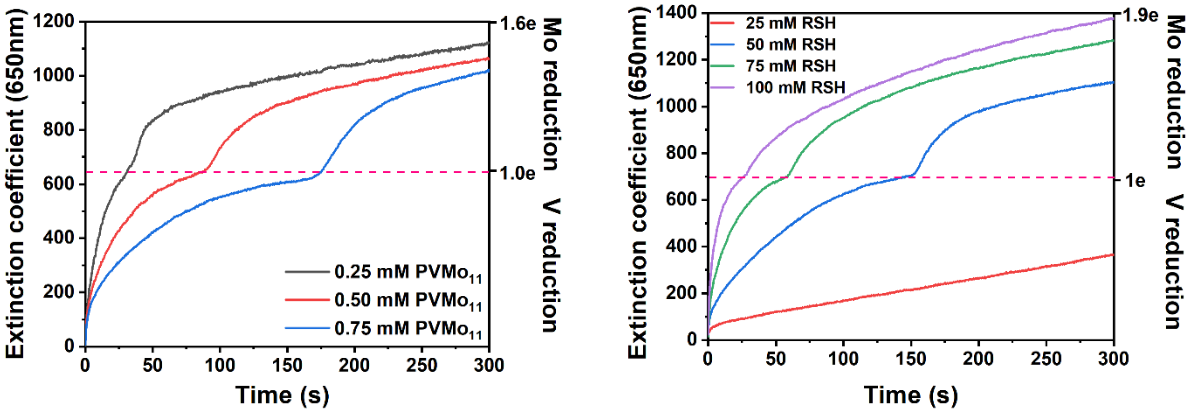


(a) (b)

**Figure S19.** (a) Kinetics of $[PVMo_{11}]^{4-}$ reduction by 50 mM RSH under Ar catalyzed by 2μM Cu(II) depend on POM concentration in pH = 2 phosphate buffer. (b) Kinetics of 0.1 mM $[PVMo_{11}]^{4-}$ reduction by RSH under Ar catalyzed by 2μM Cu(II) concentration in pH = 2 phosphate buffer.

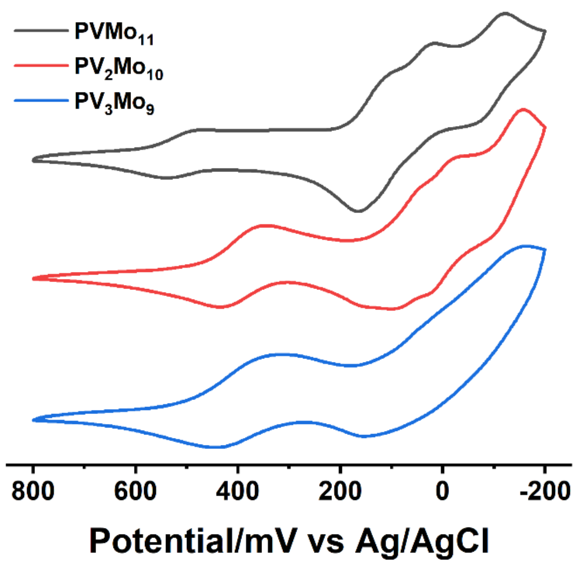


**Figure S20.** CV of $[PVMo_{11}]^{4-}$ and $[PV_2Mo_{10}]^{5-}$ in 0.1 M perchloric acid aqueous solution. Conditions: 0.5 mM POM on glassy carbon electrode. 100 mM $KNO_3$. $v$ = 100 mV s$^{-1}$, T = 298 K.

**Table S4:** The results of bulk electrolysis of $[PVMo_{11}]^{4-}$ at constant potential in pH = 2 phosphate buffer.[a]

| Potential/mV vs Ag/AgCl | Number of Coulombs | Number of electrons | Ending current ratio/% |
|---|---|---|---|
| 450 | 2.9 | 1.0 | 5 |
| 150 | 2.95 | 1.0 | 10 |
| -50 | 3.05 | 1.0 | 10 |

[a] Conditions: $[PVMo_{11}]^{4-}$ (1 mM), $KNO_3$ (100 mM), pH = 2 phosphate buffer (30 mL), at room temperature under argon.

**Table S5:** The results of bulk electrolysis of $[PV_2Mo_{10}]^{5-}$ at constant potential in pH = 2 phosphate buffer.[a]

| Potential/mV vs Ag/AgCl | Number of Coulombs | Number of electrons | Ending current ratio/% |
|---|---|---|---|
| 350 | 2.88 | 1.0 | 5 |
| 50 | 2.93 | 1.0 | 10 |
| -50 | 2.98 | 1.0 | 10 |
| -150 | 3.01 | 1.0 | 12 |

[a] Conditions: $[PV_2Mo_{10}]^{5-}$ (1 mM), $KNO_3$ (100 mM), pH = 2 phosphate buffer (30 mL), at room temperature under argon.

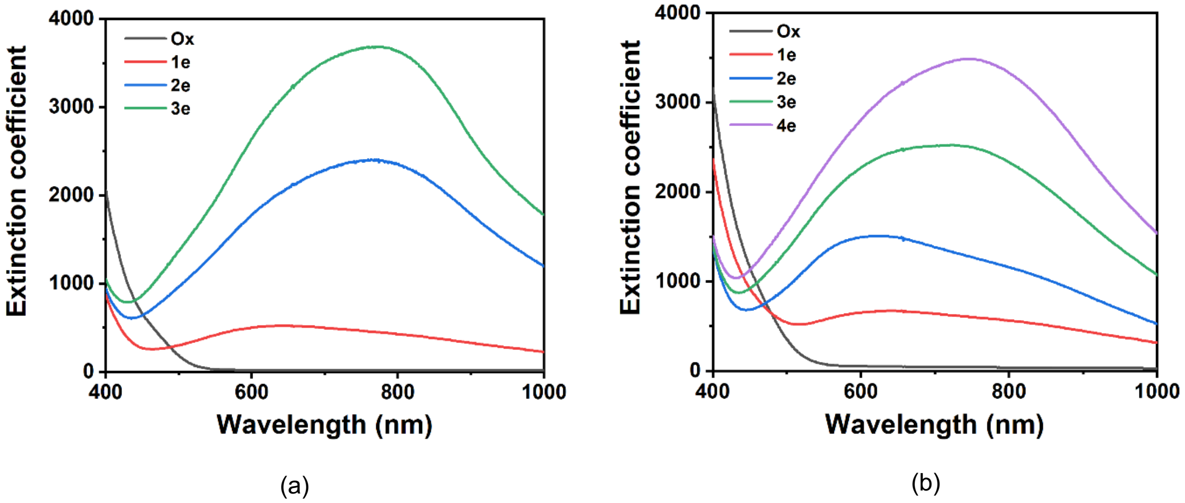


Figure S21. UV-Vis spectra for bulk electrolysis titration of (a) $[PVMo_{11}]^{4-}$; (b) $[PV_2Mo_{10}]^{5-}$ in pH = 2 phosphate buffer. The bulk electrolysis results show in Table S4 and S5.

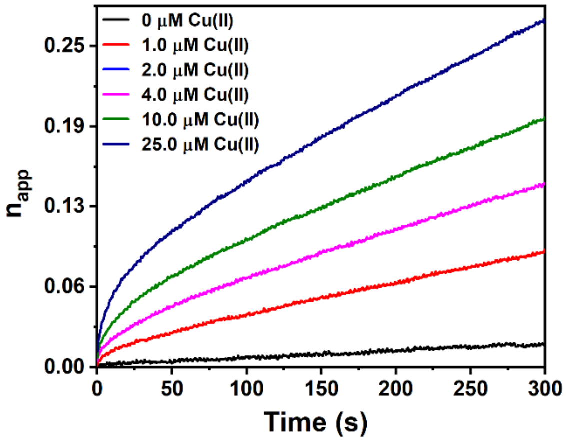


**Figure S22.** Kinetics of 0.1 mM $[PMo_{12}]^{3-}$ reduction by 50 mM RSH under Ar catalyzed by Cu(II) in pH = 2 phosphate buffer.

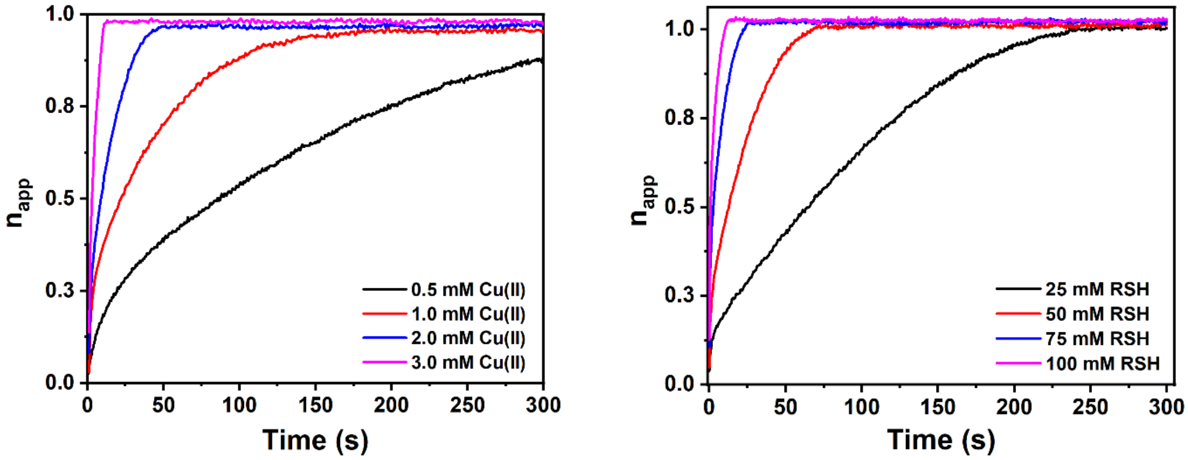


(a) (b)

**Figure S23.** Kinetics of $[PVW_{11}]^{4-}$ reduction by RSH catalyzed by Cu(II) under Ar in pH = 2 phosphate buffer. (a) Cu(II) concentration dependence with 0.1 mM POM and 50 mM RSH; (b) RSH concentration dependence with 0.1 mM POM and 2 μM Cu(II);

**Table S6:** Air-based Oxidation of 2-Mercaptoethanol Catalyzed by **PVMo**/Cu Systems[a]

| Catalyst | Conversion[b], % | TON×10$^{-2}$[c] | TOF×10$^{3}$, s$^{-1}$ [d] |
|---|---|---|---|
| **$PVMo_{11}$** | 10 | 0.30 | 5 |
| **$PV_2Mo_{10}$** | 12 | 0.36 | 6 |
| **$PV_3Mo_9$** | 16 | 0.48 | 8 |
| **$PV_4Mo_8$** | 90 | 2.7 | 45 |
| **$PV_6Mo_6$** | 100 | 3.0 | 56 |
| **$PV_4Mo_8$**[e] | 0.8 | 0.024 | 0.4 |
| **$PV_6Mo_6$**[e] | 1 | 0.03 | 0.5 |
| $Cu(ClO_4)_2$[f] | 10 | 0.04 | 0.6 |

[a] Conditions: POM (0.1 mM), $Cu(ClO_4)_2$ (0.8 mM), 2-mercaptoethanol (30 mM), acetonitrile (5 mL) at room temperature under air. [b] Conversion was measured after 100 min. [c] Turnover number (TON = moles of 2-mercaptoethanol consumed per mol of POM) was measured after 100 min. [d] Turnover Frequency, TOF = TON/(Reaction time). [e] POM (0.1 mM) without $Cu(ClO_4)_2$. [f] TON and TOF based on $Cu(ClO_4)_2$ concentration.

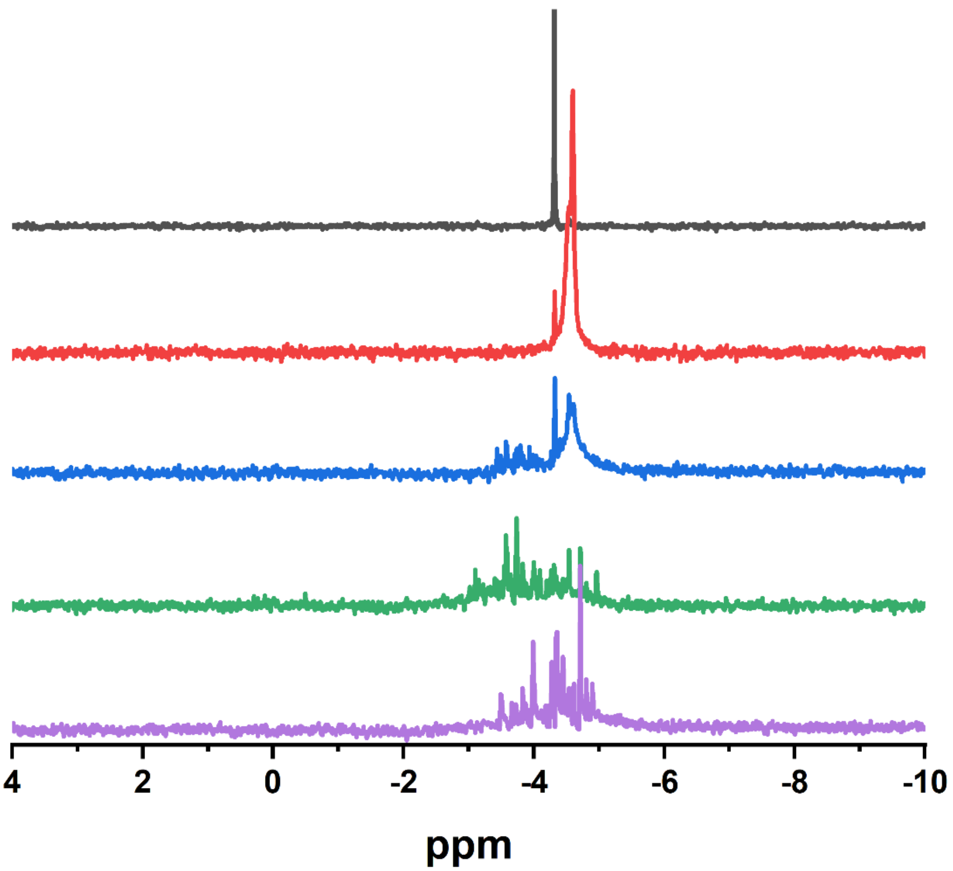


**Figure S24.** $^{31}P$ NMR spectra in acetonitrile-$d_3$ relative to 85% $H_3PO_4$ (0 ppm) of **$PVMo_{11}$**, black; **$PV_2Mo_{10}$**, red; **$PV_3Mo_9$**, blue; **$PV_4Mo_8$**, green; **$PV_6Mo_6$**, purple.

## References


(1) Tsigdinos, G. A.; Hallada, C. J. Molybdovanadophosphoric Acids and Their Salts. I. Investigation of Methods of Preparation and Characterization. *Inorg. Chem.* **1968**, *7* (3), 437–441.

(2) Yokota, T.; Fujibayashi, S.; Nishiyama, Y.; Ishii, Y.; Sakaguchi, S. Molybdovanadophosphate (NPMoV) /Hydroquinone/O, System as an Efficient Reoxidation System in Palladium-Catalyzed Oxidation of Alkenes. *J. Mol. Catal. A Chem.* **1996**, *114* (1–3), 113–122.

(3) Hamamoto, M.; Nakayama, K.; Nishiyama, Y.; Ishii, Y. Oxidation of Organic Substrates by Molecular Oxygen/Aldehyde/Heteropolyoxometalate System. *J. Org. Chem.* **1993**, *58* (23), 6421–6425.

(4) Tsigdinos, G. A. Preparation and Characterization of 12-Molybdophosphoric and 12-Molybdosilicic Acids and Their Metal Salts. *Ind. Eng. Chem. Prod. Res. Dev.* **1974**, *13*

(4), 267–274.

(5) Lu, X.; Cheng, T.; Geletii, Y. V.; Hill, C. L. Catalytic System for Aerobic Oxidation That Simultaneously Functions as Its Own Redox Buffer. *Inorg. Chem.* **2023**, *5* (62), 2404–2414.

(6) Kozhevnikov, I. V. Catalysis by Heteropoly Acids and Multicomponent Polyoxometalates in Liquid-Phase Reactions. *Chem. Rev.* **1998**, *98* (1), 171–198.

(7) Botar, B.; Geletii, Y. V.; Kögerler, P.; Musaev, D. G.; Morokuma, K.; Weinstock, I. A.; Hill, C. L. The True Nature of the Di-Iron(III) γ-Keggin Structure in Water: Catalytic Aerobic Oxidation and Chemistry of an Unsymmetrical Trimer. *J. Am. Chem. Soc.* **2006**, *128* (34), 11268–11277.

(8) Bard, A. J. *ELECTROCHEMICAL METHODS*; 2019; Vol. 2.
